# Feasibility of a Protease Activity-Based Nanosensor for Breast Cancer Screening

**DOI:** 10.1021/acsomega.5c09454

**Published:** 2026-01-19

**Authors:** Erica C. Silva, Noeli S. M. Silva, Felipe S. Soto, Júlio C. Borges, Valtencir Zucolotto

**Affiliations:** † Nanomedicine and Nanotoxicology Group, Department of Physics and Materials Science, São Carlos Institute of Physics, 117186 University of São Paulo, 400 Trabalhador São-carlense Avenue, 13569-180 São Carlos, São Paulo, Brazil; ‡ Biochemistry and Biophysics of Proteins Group, Department of Chemistry and Molecular Physics, São Carlos Institute of Chemistry, 153988 University of São Paulo, 400 Trabalhador São-carlense Avenue, 13560-970 São Carlos, São Paulo, Brazil

## Abstract

Early detection of any form of cancer increases the number of successful treatments, but a lack of predictive biomarkers is limiting progress as low tumor-to-background ratios cause current tumor biomarkers to miss early signs of disease. Activity-based nanosensors, sensing nanoparticles administered in a prodiagnostic form, tackle this limitation by generating synthetic biomarkers at disease sites and leveraging enzymatic turnover and urinary enrichment to amplify tumor signals that would otherwise remain hidden. We inform that successful activity-based nanosensor tumor accumulation requires more than engineered nanoparticles with size, surface charge, and shape to ease the entry into tumors and the use of tumor models known to have vasculature permeable to nanoparticles. We found that a protease activity-based nanosensor with a half-life amenable to distribution in organs did not accumulate in an invasive mammary tumor model characterized by diffusive and active transport entry of nanoparticles with sizes within our nanosensor size distribution. Moreover, nonspecific and off-target activations decreased the nanosensor half-life in blood, which further aided in lowering the entry into tumors, yielding no distinct increases in the tumor activity signal in urine. These findings prompted refinements of the current design criteria of activity-based nanosensors, pushing for the development of nanosensors that rely on more specific properties of tumor proteolytic activity.

## Graphical abstract

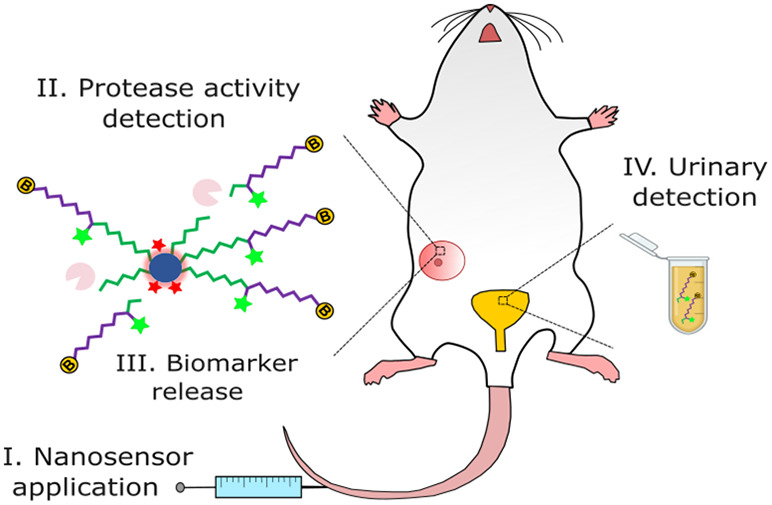

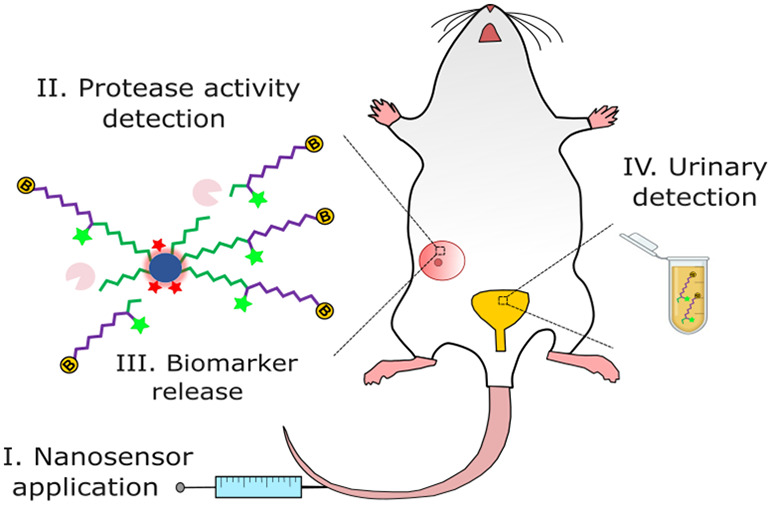

## Introduction

Cancer remains one of the major challenges in public health.^1–3^ Current detection methods perform poorly in screening settings due to intrinsically low tumor-to-background ratios that strip biomarkers of predictive power to detect the disease early.^3–5^ Accessing circulating tumor DNA (ctDNA) has yielded new potential biomarkers for cancer screening, but fragments of DNA are also released by normal cells and the amounts of shed ctDNA correlate with the rate of cancer cell death.^5–9^ Since resisting cell death is a hallmark of cancer associated with tumor growth and dissemination, blood ctDNA testing has fundamental limitations in the early detection of consequential tumors.^10–12^ Low-dose computed tomography (LDCT) identifies more suspicious masses in the lungs than standard radiography, but they are mainly benign or indolent tumors that will not become a cancer during patient’s life.^13,14^ Nevertheless, such a high false-positive rate leads to futile invasive diagnostic procedures, straining the resilience of the public health system. A typical breast tumor reaches the detection threshold between annual screenings, having already achieved a size capable of dissemination, as mammographic screenings currently identify tumor ∼5 mm in diameter.^5,15,16^ Hence, new avenues must open to address the barriers of low tumor-to-background ratios imposed on cancer screening.

Matrix metalloproteinase (MMP) activity regulates the tumor microenvironment, aiding directly in invasion and dissemination owing to its role in degradation and remodeling of the extracellular matrix.^12,17^ Activity-based nanosensors (ABNs) are nanoparticles that upon sensing protease activity release inert peptides that fairly concentrate in the urine to afford noninvasive detection.^18–23^ To tackle the low tumor-to-background issue, activity biomarker strategies rely on the ABN’s ability to accumulate in tumors and generate an amplified signal of the local proteolytic activity that is proportional to ABN delivery.^22,24,25^

Envisioning that activity-based urine testing could be combined with mammography to detect breast cancer early, we decided to develop an ABN with specificity toward MMP-2, with size, surface charge, and morphology to ease its entry into the 4T1 mammary tumor, an invasive tumor model that mirrors the triple-negative breast cancer in humans, aiming to generate a coupled pharmacokinetic description of the ABN and its associated urinary biomarker and to investigate sources of noise.^26^ From here, we expected to devise guidelines to develop a rapid test for urinary biomarker detection, informed by the dynamics of tumor signal and background noise in the blood and the amplitude of activity-based biomarker levels in urine.

We synthesized an MMP-2 ABN with the specified size and surface charge distributions as determined based on the nanocarrier data characterization and pharmacokinetics profile displayed in the blood. Surprisingly, the ABN accumulated at negligible levels in the tumor. Moreover, the level of active proteases shed by the tumor in the bloodstream rapidly cleaved the peptides on the ABN surface, decreasing the half-life of the ABN in the blood. The tumor activity signal in the urine was not statistically discernible when compared to the activity signal in the urine of healthy controls. These findings questioned the ability of ABNs to detect invasive tumors. Although blood-based approaches focus on developing methods to sensitively detect target analytes, ABNs sense the tumor proteolytic activity as a less specific property. More importantly, our results pointed to a direction in which ABNs might realize their potential as prodiagnostics by focusing on preinvasive tumors, which generally are not detected by blood ctDNA tests, and narrowing to specific proteolytic targets.^6,23,27^

## Results and Discussion

### Synthesis, Physicochemical and Optical Characterizations

The ABN was synthesized following protocols well-established in previous works.^20,21,28^ The nanosensor core was a polymeric carrier of hexaglycerol in which eight poly­(ethylene glycol) (PEG) arms bearing an amine group were built, and the entire construct assembled into nanoparticles in phosphate-buffered saline (PBS) with a spherical morphology and the mean diameter above the size cutoff for glomerular filtration, which is considered to be 5–6 nm for nanoparticles (Figure 1a).^29,30^ Size distribution measurements by transmission electron microscopy (TEM) revealed a diameter of 7.6 ± 2.2 nm and measurements of dynamic light scattering (DLS) revealed a hydrodynamic diameter of 9.7 ± 1.6 nm under physiological conditions, confirming that a potential ABN could escape renal clearance from the bloodstream (Figures 1a–c and S1a).

**Figure 1:**
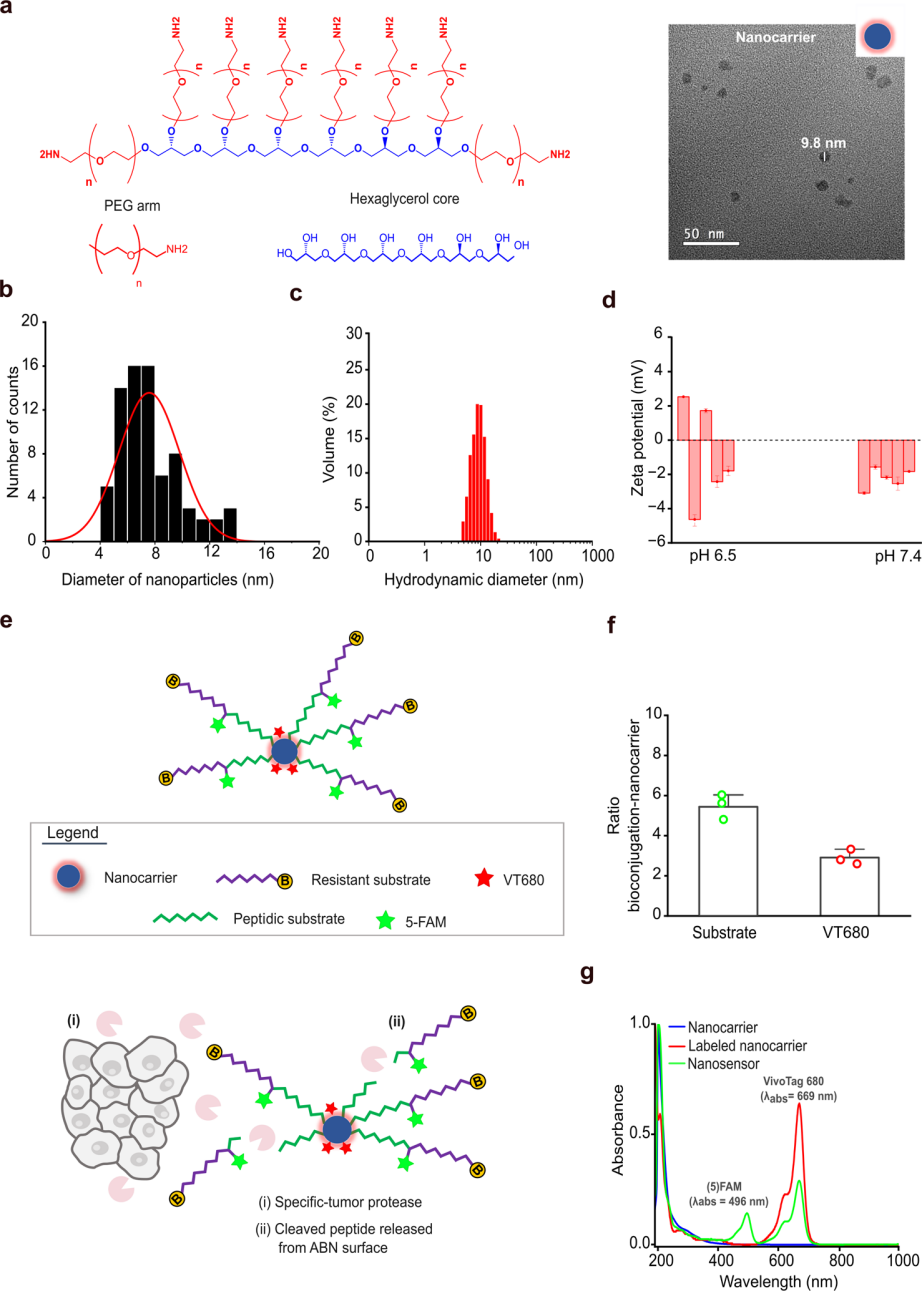
Design of the ABN to detect invasive breast cancer. (a) Molecular structure of a nanocarrier. Schematic and TEM images showing its assembly into nanoparticles. The scale bar is as indicated in the image. PEG, poly­(ethylene glycol). (b) Nanocarrier size distribution was determined by quantifying the diameter of the spherical particles in TEM micrographs. (c) Hydrodynamic size distribution of the nanocarrier measured in PBS at pH 7.4 and 37 °C. (d) Measurements of the zeta potential of nanocarriers dispersed in saline at pH 6.5 and pH 7.4 at 25 °C. (e) Schematic depicting a functionalized nanocarrier to serve as the ABN, displaying the peptidic structure made of an MMP-sensitive substrate (green) and a protease resistant peptide (purple). Active proteases are expressed by cancer and normal cells in the tumor microenvironment (i). Delivered ABNs signal tumor proteolytic activity by releasing inert peptides that diffuse out of the tumor to be detected in urine as activity-based biomarkers (ii). B, biotin; 5-FAM, 5-carboxyfluorescein; VT680, VivoTag. (f) Valence of bioconjugation of the peptide and VT680 dye on the ABN surface, indicating that the arms reacted with peptides and VT680 in a 5:3 ratio. (g) UV–visible spectra of the labeled nanocarrier and ABN, showing bands associated with 5-FAM and VT680 light absorption, confirming the construction of the ABN.

These nanoparticles showed a neutral surface charge with zeta potential distributions toward negative values −0.8 ± 3.3 mV under physiological conditions with pH 7.4 and −2.2 ± 0.6 mV under acidic conditions with pH 6.5, matching the physiological bloodstream and the acidic tumor environments (Figures 1d and S1b). Such findings indicated that the nanoparticles avoid nonspecific interactions with negatively charged entities in the blood, such as phagocytes, which implies a longer residence time.^31–33^ The absolute values of the zeta potential indicated that nanocarrier dispersions have tendencies toward agglomeration and aggregation, but DLS measurements showed that formulations are stable under physiological conditions for 48 h after preparation (Figure S1c).

We elected a synthetic peptide (TIAH–LH) with specificity toward MMP-2, a protease involved in tissue invasion and intravasation of cancer cells, to synthesize the ABN (Figures S2 and S3).^17,34^ A cleavage-resistant glutamate fibrinopeptide B, a peptide rapidly excreted in the kidneys, was synthesized in tandem with the peptide substrate to serve as the signal source to qualify as a tumor activity biomarker (Figure S3a).^24,28,35^ To permit ABN prototyping attributes, 5-carboxyfluorescein (5–FAM) was flanked in the tandem peptide to aid in the estimation of the blood half-life and urinary enrichment, and biotin was conjugated to the N-terminal to primarily ease the development of a noninvasive detection test (Figures 1e and S4). The valency of conjugation revealed minimum batch-to-batch variation, yielding ∼5 tandem peptides and ∼3 VT680 labels per nanocarrier, which implies that the eight arms were, on average, occupied (Figures 1e,f and S3b,c). UV–visible absorbance spectra indicated that the ABN was successfully synthesized by displaying specific bands associated with the maximum light absorption of 5-FAM and the infrared VT680, which was conjugated directly onto the ABN surface (Figures 1g and S3a). Fluorescence emission spectra confirmed that the ABN was optically active and that the dyes did not cross-talk destructively (Figure S3d).

### In Vitro MMP-2 Activity and ABN Toxicity Assessment

Probes were synthesized by using a fluorescence resonance energy transfer (FRET) substrate conjugated onto the nanocarrier surface at varying distances to study substrate presentation effects on MMP-2 catalytic efficiency (Figure S5). While probes with substrates at a distance of 1.5 Å from the nanocarrier surface developed signals at noise levels, probes synthesized with substrates at 95.2 Å from the core exhibited lowering fluorescence signals over probe concentrations greater than 1.5 μM due to inner filtering effects, causing loss of linearity in the initial reaction velocity (Figures S6 and S7). Probes with substrates presented at 32.5 and 53.4 Å generated signals that allowed to estimate the catalytic efficiency of full-length MMP-2 at 32 nM. Catalytic efficiency (∼ 10^4^ M^–1^ s^–1^) on probes with substrates presented at 32.5 Å was aligned with the calculation on the substrate immobilized on solid surfaces and subjected to the catalytic domain of MMP-2 at 50 nM.^36^ On the probe with substrates presented at 53.4 Å, the catalytic efficiency decreased by an order of magnitude (Figure 2a–c). We decided to present the substrate at 53.4 Å from the ABN surface as the effect of steric hindrances on MMP-2 ability to activate probes was negligible.

**Figure 2:**
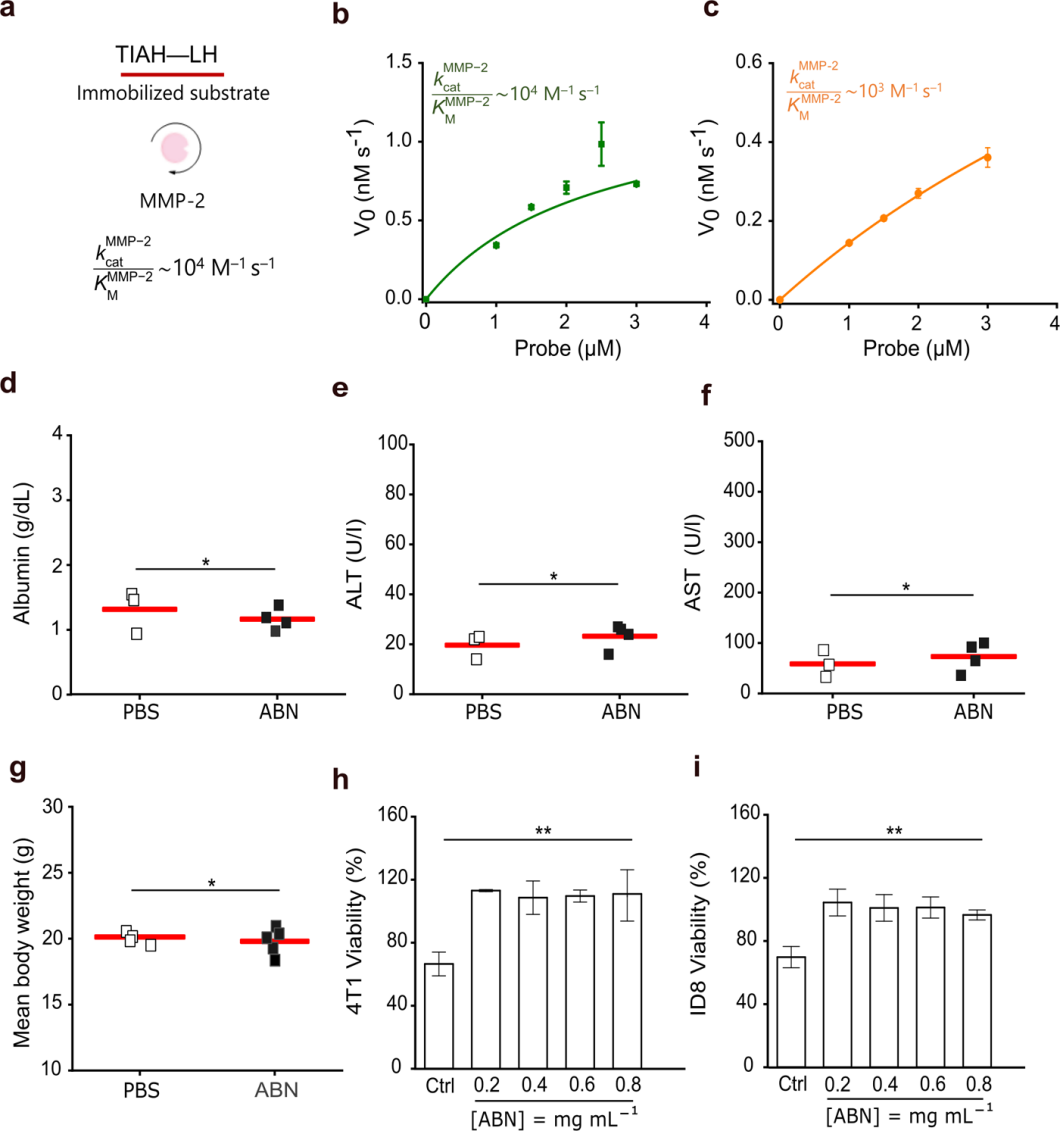
Catalytic efficiency of MMP-2 on fluorogenic probes and assessment of the ABN toxicity. (a) Schematic showing the catalytic efficiency of MMP-2 on TIAH–LH, immobilized on the surface of microplate wells and incubated with the 50 nM catalytic domain for 2 h at 37 °C. Initial cleavage velocity of the substrate conjugated onto the nanocarrier surface at (b) 32.5 Å and (c) 53.4 Å from the core, highlighting the catalytic efficiency. The lines represent the Michaelis–Menten model fit to the data. mean ± s.e.m. is denoted by a horizontal line with error bars; *n* = 2 wells per probe concentration; *N* = 3; *R* ^2^ = 0.87; *R* ^2^ = 0.99. *K*_cat_, catalytic constant; *K*_M_, Michaelis–Menten constant. (d–g) Blood levels of hepatotoxicity markers of the mice injected with the ABN were equal to the levels found in the controls. The mean body weight remained stable for 5 d after ABN injection. Each square represents one mouse, and the mean value is denoted by the red bar. The Shapiro–Wilk test is followed by the unpaired two-tailed *t*-test; *P > 0.05. ALT, alanine aminotransferase; AST, aspartate aminotransferase. (h, i) Viability of 4T1 and ID8 cells after incubation with the ABN at different concentrations for 24 h. mean ± s.e.m. is denoted by a horizontal line with error bars; *n* = 2–3 wells per condition; *N* = 3; one-way ANOVA with Bonferroni multiple comparisons; **P < 0.05. Ctrl, positive control: acetaminophen at 25 mM in cell culture media.

Albumin, alanine aminotransferase, and aspartate aminotransferase levels in plasma remained at control levels 24 h after a high dose 2 × 10^9^ mol of the ABN in 200 μL of PBS was intravenously injected into mice. We did not also observe changes after tracking body mass for 5 days after injection (Figure 2d–g). Ovarian ID8 and breast 4T1 tumor cells remained healthy after 24 h of incubation with a formulation of the FRET ABN at different concentrations when compared with positive controls (Figures 2h,I and S8). These specific findings demonstrated that the ABN was safe for administration and could cause no harm to potential target organs. Nevertheless, long-term toxicity has yet to be evaluated for a comprehensive assessment.

### Pharmacokinetics and Amplification of Urinary Signals

The pharmacokinetics of the ABN in the blood described by its distribution and elimination half-lives revealed that the circulating ABN had a residence time to distribute across organs and accumulate in tumors (Figure 3a). By assessing the kinetics of the concentration of nanocarriers in plasma, we found that the elimination half-life of the ABN, which depends on renal filtration and hepatobiliary clearance rates, was longer than the half-life of the nanocarrier, pointing to peptides on the ABN surface that affect the hydrodynamic diameter and surface charge, decreasing the clearance rate of the ABN from the blood (Figure 3a,b). ABN breakdown by blood proteases was evident by comparing signals from peptides on the ABN surface and free peptides in the bloodstream, both of which displayed similar profiles, showing that a relevant detection threshold can be challenging for ABNs (Figure 3c).^24,28,37^ In addition, we observed that an amount of nanocarriers was excreted in the kidneys, which implies that the surface charge plays a role in the renal absorption of ABNs with diameters near or below the filtration pore size (Figure 3d).^30,31^

**Figure 3:**
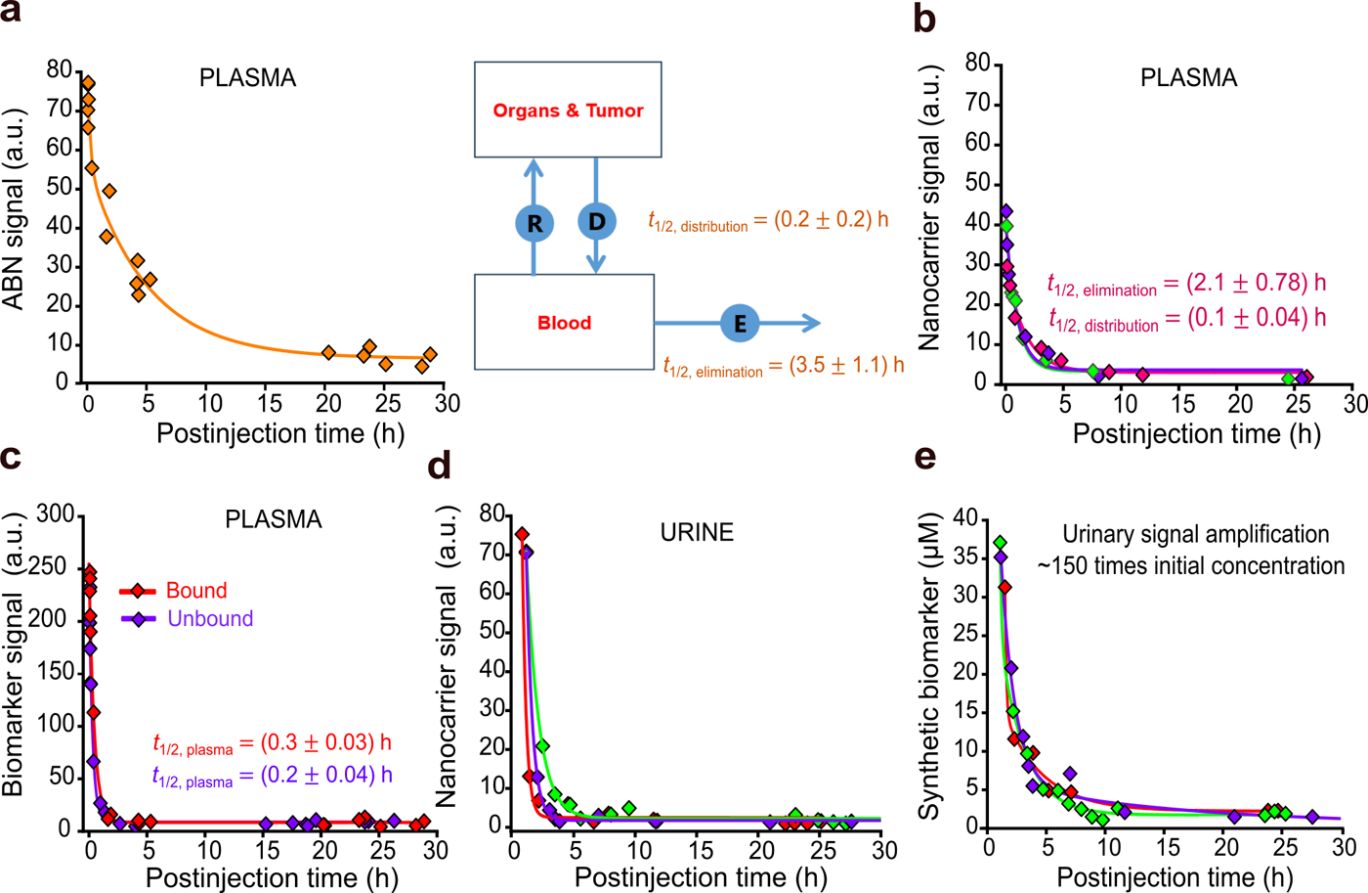
Pharmacokinetics of the ABN and the synthetic biomarker. (a) Kinetics of ABN concentration in plasma predicted by a two-phase exponential decay model. Schematic showing the pharmacokinetics of a two-compartment model. *n* = 6 mice; *R* ^2^ = 0.97. Values are represented as mean ± s.e.m. *D*, distribution. *E*, elimination. *R*, redistribution. (b) Plasma half-lives of nanocarriers were estimated by fitting a two-phase exponential decay model to the data. *n* = 3–5 mice per curve; *R* ^2^ = 0.99. Estimated half-lives of one set are shown in the plot area. Values are represented as mean ± s.e.m. *t*_1/2_, half-life. (c) Kinetic profile of the synthetic biomarker on the ABN surface and free peptides in plasma. Lines represent the fit of a one-phase exponential decay model. *n* = 3–5 mice per curve; *R* ^2^ = 0.99, biomarker; *R* ^2^ = 0.95, peptide. Values are represented as mean ± s.e.m. (d) Kinetic measurements of nanocarrier signals in urine show that part of its population underwent glomerular filtration. *n* = 1 mouse per curve. (e) Kinetics of the concentration of activity-based biomarkers in urine and the signal amplification estimation. *n* = 1 mouse per curve.

Bearing in mind that urinary enrichment equally affects the tumor signal and background noise in the blood, we estimated a signal amplification of ∼2 orders of magnitude compared with the maximum concentration in the blood after injection of the free peptide dose (Figures 3e and S9). When increasing the size of ABNs imposes undesired trade-offs between the size and clearance rate from the blood, the ABN filtered into the urine generated noise that imposes higher thresholds on analytical tools to screen urine for tumor-cleaved peptides.^11^ Here, the dynamic range rather than the sensitivity determines the test reliability. Paper-based tests that usually have short dynamic ranges have limited power to advance early cancer detection without strategies of broadening their dynamic ranges and to take advantage of the ABN and biomarker pharmacokinetics (Figure S4).^35,38^ To further our investigation, we tested the ABN in a mouse model of triple-negative breast cancer (Figures S10 and S11).^26^

### ABN Performance in a Challenging Proteolytic Activity Environment

To serve as prodiagnostic agents, ABNs must circulate in stealth, home to tumors, and probe the microenvironment to generate signals of aberrant activity that are readily measurable in urine. Nanomedicines for cancer care are designed to accumulate in tumors to deliver therapeutics directly to cancer cells or sample the microenvironment to generate informative signals of ongoing malignant processes.^33,39^ Specifically, ABNs have to probe the areas of exacerbated proteolytic activity. We challenged our ABN to profile breast tumors in a mouse model that recapitulates the formation of the tumor microenvironment in humans as coopted immune cells populating the surroundings of cancer cells secrete most of active proteases (Figure S10a,b).^17,26^ The ABN was injected into mice bearing a four-week-old tumor expressing elevated levels of MMP-2 confirmed by Western blot analysis of the anti-MMP-2 in lysates that showed a specific band detected for MMP-2 at a molecular weight of 75 kDa (Figure S10c,d).

The tumor proteolytic activity altered ABN flow through the body, as its kinetics in the plasma from tumor-bearing mice behaved distinctively compared with the healthy counterparts, implying that the peptides on the surface of the ABN were cleaved by blood and tumor-shed proteases (Figure 4a). The kinetic profile of the activity biomarker also showed signs of high protease activity in the blood, as the plasma half-life of the activity biomarker decreased compared with the half-life in healthy mice (Figure 4b). After urine separation by polyacrylamide gel electrophoresis, fluorescent images of the gels further confirmed that the ABN in the urine of diseased mice had lost all peptides, while the ABN in healthy mice had not (Figure S12).

**Figure 4:**
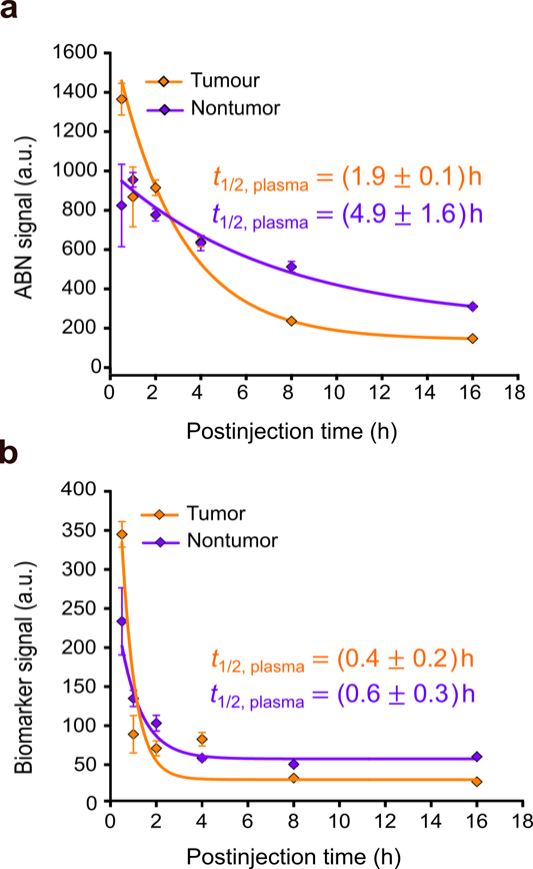
ABN performance in the 4T1 mammary tumor model. (a) Kinetics of the ABN signal in the plasma of mice bearing a four-week-old tumor. The lines denote the behavior predicted by a one-phase exponential decay model. Values are represented as mean ± s.e.m; *n* = 2–4 mice; *R* ^2^ = 0.99, tumor; *R* ^2^ = 0.98, nontumor. (b) Kinetics of the biomarker signal in the plasma of tumor-bearing mice versus normal counterparts, indicating the exacerbation of protease activity in the blood. Lines denote the kinetic behavior by a one-phase exponential decay model. Values are represented as mean ± s.e.m.; *n* = 2–4 mice; *R* ^2^ = 0.78, tumor; *R* ^2^ = 0.74, nontumor.

The biodistribution of the ABN across the tumor alongside the major organs demonstrated negligible levels of the localized ABN in the tumors compared with the background signal in tumors extracted from uninjected mice (Figure 5a). Furthermore, the ABN was absorbed into the kidneys where the signal intensity peaked at 0.25 h after injection, and a significant signal intensity in the spleen at initial time points demonstrated that the ABN was subjected to clearance by the mononuclear phagocyte system (Figure S13).^31^ Urinary activity signal measurements were from ABN activation outside the tumor by native blood and tumor-shed proteases, but the background generated by this off-tumor activation did not suffice to differentiate diseased mice from healthy controls (Figure 5b).

**Figure 5:**
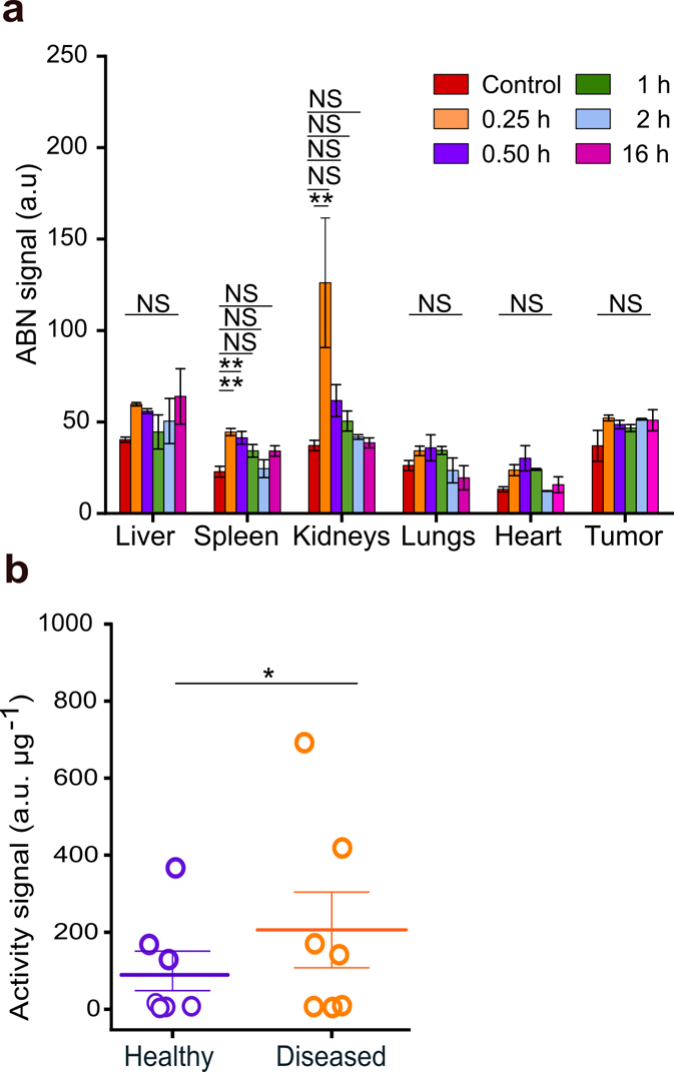
Localized ABN signals and urinalysis. (a) Distribution of the ABN in the major organs and tumor accumulation at postinjection time points, indicating a low ABN signal in tumors and demonstrating rapid clearance of the ABN with a size below renal cutoff at 0.25 h. mean ± s.e.m. is represented by a horizontal line with error bars; the Shapiro–Wilk test followed by one-way ANOVA with multiple comparisons; **P < 0.05. NS, not significant. (b) Signal of urinary activity of healthy versus diseased mice normalized to the total protein content. Urine was collected up to 1 h after ABN application. mean ± s.e.m. is denoted by a horizontal line with error bars; Mann–Whitney *U* test; *P > 0.05.

No signal was generated from the mammary tumor microenvironment. The biodistribution analysis showed no significant accumulations of the ABN in tumors. Our ABN was synthesized to display size and surface charge distributions amenable to passive diffusion similar to ABNs from the literature.^19,24,28^ We relied on the notion that engineered nanoparticles extravasate tumor vasculature through open pores.^29,33,40^ Nevertheless, this rationale is the basis of innovative approaches for cancer care that have been translated poorly to the patients’ bed.^29,33^

The breakdown of the ABN by blood and tumor-shed proteases changed the kinetics of ABN concentration in the blood to display a profile that prevented the ABN from generating signals from tumors as most circulating ABNs lost the prodiagnostic form within ∼30 min (Figure 4a). Depending on the level of active proteases in the blood, such results imply that ABNs unable to evade off-tumor activation in the blood can hardly serve as prodiagnostic agents as proteases’ catalytic efficiency is orders of magnitude higher than the rates at which ABNs can cross tumor wall vessels.^24,28^ To tackle this issue, a previous study designed ABNs with a substrate with no specificity toward blood protease thrombin to reach higher target specificity.^24^ Nevertheless, ABNs have been designed with unshielded sensing substrates that can generate noise in the bloodstream due to overlapping substrate specificity among proteases, making the task to assign specific-cancer proteases to the cleavage signatures in urine cumbersome.^27,36,41^

The prodiagnostic power of ABNs for cancer screening was showcased by a study using two models of lung cancer driven by common mutations to initiate tumorigenesis, mirroring the development of lung cancer in humans and giving rise to a microenvironment populated by immune cells that secrete activated proteases.^17,20,22^ ABNs were synthesized with 14 mass-coded substrates by isobaric labeling to allow differentiation based on cleavage pattern by mass spectrometry analysis of urine. Herein, they were intratracheally instilled to directly access tumors’ modules, taking advantage of the lungs as a transport surface that connects their contents with the circulatory system, thereby bypassing the blood nonspecific and off-target activations. The cleavage patterns in the urine allowed correct identification of the two types of lung cancer, discriminating them from a common lung inflammation.^20^ The multiplexing feature covered activities of proteases of different catalytic classes and raised comprehensive cleavage profiles in the urine, which when recognized by a classification algorithm allowed the test to achieve a high sensitivity that outperformed a ctDNA blood test applied in a similar genetic-driven lung cancer model by comparing the total burden, 2.8 mm^3^ versus 7.1 mm^3^, when tumors were detected.^42^

Activity-based biomarkers have the potential to outperform blood DNA tests, which have poorly performed in detecting cancer at early stages, as shown by studies with humans (Table S1). The estimated sensitivities have been as small as 27% when one test combined with an imaging method was used to screen women not known to have cancer.^6,7,20^ Knowing that the majority of the cells in preinvasive tumors are normal and the tumor-promoting inflammation by protease-secreting immune cells enables tumorigenesis, ABNs should be tuned to identify tumors at preinvasive stages, prior to exacerbation of potent proteolytic cascades by cancer cells.^17^ Combining these facts with our findings enforced that the controlled entry of ABNs into tumors demands new design criteria to maximize tumor signal and minimize off-tumor noise, which are met by assessing the mechanisms of tumor entry to modulate the ABN propriety of crossing the tumor vasculature barrier, decoupling protease activity to develop specific sensing moieties to hinder off-tumor activation, and deriving mathematical frameworks on characterization data to guide rational design.^24,28,29^

## Conclusions

Our findings reminded us that we need more studies that focus not only on showcasing innovative ABNs, but also on strengthening the fundamental knowledge of how these devices interact with the tumor microenvironment using mechanistic models in which ABNs’ data characterization and tumor models’ transport properties are built to predict the nanosensor accumulation in tumors and the amplitude of tumor activity signal in urine. The noise that ABNs generates while querying the organism for active tumors confounds the tumor signal, making it undiscernible if cancer cells have already exacerbated protease activation cascades. To intercept cancer before dissemination, ABNs should be designed to detect preinvasive tumors in which we might identify target proteases, whose activity can be specifically associated with cancer cell's presence, narrowing precisely to tumors that current and emergent diagnostic methods have fallen short to detect. ABNs might realize their full potential as prodiagnostic agents by focusing on detecting tumors in their earlier stages of tumorigenesis when the landscape of protease activation is less intricate, thus limiting the background generation while easing the identification of potential target protease.

## Experimental Section

Full experimental details are available in the Supporting Information.

### Animal Studies

All experiments were reviewed and approved by the Animal Care Committee of the São Carlos Physics Institute (protocol no. 7775180322) and conducted in compliance with national policies issued by the National Council for Control of Animal Experimentation.

### Nanocarrier Physicochemical Characterization

Size and size distribution were evaluated by TEM image analysis (JEOL, JEM-2100-JEOL) and DLS measurements (Malvern, Zetasizer Nano ZS90). Surface charge was characterized by electrophoresis with laser Doppler and zeta potential measurements (Malvern, Zetasizer Nano ZS90).

### Nanosensor Synthesis and Optical Characterization

The nanosensor was prepared via *N*-hydroxysuccinimide and maleimide conjugation chemistries. The molecular structure and fluorescence activity were characterized by UV–visible absorbance spectroscopy (Hitachi, High Technologies U-2900) and fluorimetry (Agilent Technologies, Cary Eclipse).

### Fluorogenic Probe Synthesis and Optical Characterization

The FRET substrate was synthesized by Thermo Fischer Scientific. Probes were synthesized in house via *N*-hydroxysuccinimide and maleimide chemistries using a 500-fold molar excess of the substrate to guarantee full nanocarrier’s arm occupation. UV–visible and fluorescence spectra were recorded in an MMP-2-specific buffer.

### In Vitro MMP-2 Activity and Biomarker Release Kinetic Assays

Fluorogenic probes dispersed in MMP-2-specific buffer at different concentrations were incubated with 32 nM mouse recombinant MMP-2 (R&D Systems, 924-MP-010) in a final volume of 100 μL to monitor fluorescence emission at 398 nm and measure dequenching at 37 °C (VarioskanTM LUX 3020–197, Thermo Fisher Scientific).

### Cell Culture

Mouse breast cancer cell 4T1 was cultured in ATCC-formulated RPMI medium supplemented with 10% fetal bovine serum and mouse ovarian surface epithelial cell ID8 in high glucose DMEM supplemented with 4% fetal bovine serum, 5 μg mL^–1^ insulin and transferrin, and 5 ng mL^–1^ sodium selenite. Both were supplemented with 1% penicillin–streptomycin.

### Toxicity Assessment

Hepatotoxicity biomarkers were measured using a biochemistry analysis platform (Labmax 240 premium, Labtest) in plasma samples from 100 μL blood draws 24 h after female C57BL/6 mice were administered through the tail vein with 2 × 10^9^ mol of the ABN in 200 μL of PBS.

### Expression Levels of MMP-2 in the 4T1Mammary Tumor Model

The identity and similarity of the human and mouse MMP-2 sequence were searched using Clustal Omega suggesting a cross-reaction between the antihuman MMP-2 antibody and mouse MMP-2. Standards methods for Western blotting were used for the detection of MMP-2 from mammary tumor lysates.

### Pharmacokinetic Studies

Female C57BL/6 mice were injected through the tail vein with the ABN (200 μL, 3 μM) in phosphate buffer or with free peptides (200 μL, 2.4 μM) in PBS. The blood was drawn from the subclavian vein, and mice were placed in individual custom cages for urine collection. Plasma was diluted 5-fold and urine was diluted 10-fold in PBS before fluorescence recordings.

### Breast Cancer Orthotopic Model Studies

Forty microliters of a suspension at 2.5 × 10^5^ 4T1 cells per mL in PBS were unilaterally injected into the fourth mammary fat pad of female BALB/c mice. The control group was injected with 40 μL of PBS only. All mice were injected through the tail vein with the ABN (150 μL, 5 μM) in phosphate buffer 28 days after tumor cell or sham injections, for distribution measurements and urinalysis.

### Preparation of a Paper-Based Lateral Flow Assay

The lateral flow strips were 25 mm long and 4 mm wide and printed with two streptavidin (Roche, 11721674001) test lines and one goat antimouse IgG (EMD Millipore, AQ127) control line. Activity-based biomarker nanoparticle–antibody conjugates were prepared using forty-nanometer-gold nanoparticles (Abcam, ab154873) and human recombinant monoclonal fluorescein antibodies (Abcam, ab206509). Assays were performed placing the strips into a separate microcentrifuge tube filled with biomarker solutions to be immunochromatographed for 15 to 30 min. The strip was then immersed in a tube filled with 200 μL of washing buffer until the color developed in the test and control lines. All strips were imaged with a Galaxy A14 5G phone camera, and all images were analyzed with ImageJ.

### Statistical Analysis

Statistical analyses were performed using PSPP (GNU, 1.6.2). Mean comparisons were performed with the *t*-test and one-way analysis of variance (ANOVA). *P* values less than 0.05 were considered to be significant. Sample sizes, post hoc statistical tests, and reproducibility of experiments are detailed in the figure legends.

## Supplementary Material



## Notes

Full experimental section; additional information and details regarding auxiliary experiments, procedures, analyses, discussions of MMP-2 identification as a target, substrate selection, and DNA blood testing for cancer screening (PDF)

## Abreviations

ABN: activity-based nanosensor
ANOVA: analysis of variance
CPQ2: proprietary fluorescence suppressor
LDCT: low-dose computed tomography
ctDNA: circulating tumor DNA
DLS: dynamic light scattering
FAM: carboxyfluorescein
FRET: fluorescence resonance energy transfer
MMP: matrix metalloproteinase
PBS: buffered phosphate saline
PEG: poly­(ethylene glycol)
s.e.m: standard of the mean
TEM: transmission electron microscopy.
