# Supplementary material for: Feasibility of a Protease Activity-Based Nanosensor for Breast Cancer Screening
Source: ACS Omega. 2026 Jan 19;11(4):5685–93. doi: 10.1021/acsomega.5c09454 (PMC12878773; doi:10.1021/acsomega.5c09454)
Supplement: Supplementary file 1 [file ao5c09454_si_001.pdf]

Supporting Information for

Feasibility of a Protease Activity-Based Nanosensor for Breast Cancer Screening

Erica C. Silva*,† Noeli S. M. Silva‡, Felipe S. Soto†, Júlio C. Borges‡ and

Valtencir Zucolotto*,†

†Nanomedicine and Nanotoxicology Group, Department of Physics and Materials Science, São Carlos Institute of Physics, University of São Paulo, 400 Trabalhador São-carlense Avenue, 13569-180 São Carlos, Brazil.

‡Biochemistry and Biophysics of Proteins Group, Department of Chemistry and Molecular Physics, São Carlos Institute of Chemistry, University of São Paulo, 400 Trabalhador São-carlense Avenue, 13560-970 São Carlos, Brazil.

*Corresponding author: erica.corina@alumni.usp.br.
zuco@ifsc.usp.br.

The file includes:

Experimental Section
Figures S1 to S13
Table S1
Supporting References

Experimental Section

Animal Studies

Tumor-bearing mice were monitored by institutional veterinarians responsible for animal welfare. For all invasive procedures, the mice were anesthetized with isoflurane (Cristália, Isoforine®), kept in a separate cage, and observed during recovery. To compare activity signals in urine, group size was not determined based on statistical power analysis, but instead roughly according to the number of mice housed per cage (n = 5). The investigator was not bound by group allocation. The samples were allowed to have at least three biological replicates and the exclusion of data was based on ABN injection, urine production, and blood collection failures at experimental time points.

Nanocarrier Physicochemical Characterization

For TEM size distribution evaluation, 4 μL of nanocarrier at 0.1 mg mL^{-1} in PBS was pipetted onto lacey copper grids, which were blotted to remove any excess samples and stored in a desiccator at room temperature before use. Imaging was performed on a JEM-2100-JEOL microscope operated at 200 kV and recorded on a Gatan 833J43N CCD camera. The images were representative of ten different locations on the sample grid. TEM micrographs were loaded into ImageJ (Fiji) to quantify the size of spherical particles. Measurements of dynamic light scattering were performed (Malvern, Zetasizer Nano ZS90) in dispersions at 2 mg mL^{-1} in PBS at 37 °C over time to assess stability. The surface charge of the nanocarrier dispersed at 2 mg mL^{-1} in saline (10 mM NaCl with pH 6.5 or 7.4) at 25 °C was characterized by electrophoresis with laser Doppler and zeta potential (Malvern, Zetasizer Nano ZS90). The material refractive index of 1.40, the aqueous medium refractive index of 1.334 and the viscosity of 0.713 cp were used for the volume-weighted size calculations. A solution of 150 kDa dextran at 2 mg mL^{-1} in PBS was characterized to validate the procedure. A viscosity of 0.891 cP, a dielectric constant of 78.6 and a Henry function of 1.5 were used for the calculations of the zeta potential distribution. All experiments were completed three times using aliquots from different dispersions.

Nanosensor Synthesis and Optical Characterization

The peptides were synthesized by Thermo Fisher or CPC Scientific. The nanocarrier was dispersed in conjugation buffer (50 mM carbonate/bicarbonate and pH 8.5) and mixed with a 10-fold molar excess of VT680 dye (Revvity, NEV11119) suspended in dimethylsulfoxide (DMSO). The mix was incubated at room temperature for 1 h before purification by size exclusion chromatography (Bio-Rad, Bio-Gel P-30) and recovery in activation buffer (10 mM Na_2HPO_4, 150 mM NaCl, pH 7.5). The labeled nanocarrier was activated with a 100-fold molar excess of NHS-PEG12-Maleimide (Thermo Scientific, 22112) in DMSO for 30 min at room temperature. Cross-linker excess was removed using size exclusion columns equilibrated with peptide conjugation buffer (10 mM Na_2HPO_4, 150 mM NaCl, 3 mM EDTA, pH 7). The activated nanocarrier was reacted with a 10-fold molar excess of peptide suspended in conjugation buffer for 1 h following the addition of L-cysteine solution at 255 mM to quench free maleimide groups and purification. The ABN was stored in PBS or phosphate buffer (5 mM Na2HPO4, pH 7.2) at 4 °C for characterization or use in in vivo experiments. The UV-visible absorbance and fluorescence spectra of the peptide solution in the conjugation buffer, the labeled nanocarrier in the activation buffer, and the ABN in PBS were recorded on a UV-visible spectrophotometer (Hitachi, High-Technologies, U-2900) and a fluorimeter (Agilent Technologies, Cary Eclipse).

Fluorogenic Probe Synthesis and Optical Characterization

The fluorescence resonance energy transfer (FRET) substrate was synthesized by Thermo Fisher Scientific. The nanocarrier in activation buffer (10 mM Na_2HPO_4, 150 mM NaCl, pH 7.5) was reacted with a 500-fold molar excess of N-succinimidyl iodoacetate (Thermo Scientific Pierce SIA, 22349) or NHS-PEG_n-Maleimide [(n = 6 (32.5 Å), 12 (53.4 Å), 24 (95.2 Å)); Thermo Scientific; 2210, 22112, 2214] crosslinkers for 30 min at room temperature before purification and recovery in peptide conjugation buffer (10 mM Na_2HPO_4, 150 mM NaCl, 3 mM EDTA, pH 7.0). The crosslinker-derivatized nanocarrier was reacted with a 64-fold molar excess of FRET substrate suspended in DMSO and peptide conjugation buffer (1:1 by volume). The reactions were allowed to continue for 1 h following the addition of L-cysteine to block free maleimide groups. The fluorogenic probes were purified in an MMP-2-specific buffer [50 mM Tris-Cl (pH

7.5), 10 mM $CaCl_2$, 150 mM NaCl] and stored at 4 °C before use or optical characterization. UV-visible and fluorescence spectra of the probes were recorded in an MMP-2-specific buffer.

In Vitro MMP-2 Activity and Biomarker Release Kinetic Assay

Fluorogenic probes dispersed in MMP-2-specific buffer at 0 μM, 1 μM, 1.5 μM, 2 μM and 3 μM were incubated with 32 nM mouse recombinant MMP-2 (R&D Systems, 924-MP-010) in a final volume of 100 μL in a 96-well plate to monitor fluorescence at 328/398 nm (ex/em) and measure dequenching at 37 °C (Varioskan TM LUX 3020 -197, Thermo Fisher Scientific). For unit conversion, 1 μM of FRET substrate in reaction buffer was hydrolyzed and its correspondent fluorescence signal was monitored until a plateau was reached, which indicated complete hydrolysis. The parameters of the Michaelis-Menten model were estimated by assessing initial cleavage velocities.

Cell culture

The 4T1 mouse breast cancer cell line was purchased from Rio de Janeiro Cell Bank (BCRJ, 0022) and cultured in ATCC-formulated RPMI medium supplemented with 10% fetal bovine serum. The ID8 mouse ovarian surface epithelial cell, which can acquire malignant characteristics through prolonged passages in vitro, was purchased from EMD Millipore (Millipore, SCC145) and cultured in high glucose DMEM supplemented with 4% fetal bovine serum, 5 μg mL^{-1} insulin and transferrin and 5 ng mL^{-1} sodium selenite. Both cell cultures were supplemented with 1% penicillin-streptomycin and incubated at 37 °C with 5% CO_2. Mycoplasma tests were performed by the corresponding proprietary cell bank.

Toxicity Assessment

Hepatotoxicity biomarkers were measured in plasma samples free of hemolysis from 100 μL blood draws 24 h after mice were administered through the tail vein with 2 × 10^9 mol of the ABN in 200 μL of PBS (the control mice received only PBS). Measurements were performed using a biochemistry

analysis platform (Labmax 240 premium, Labtest) and standard methods (albumin: colorimetric–bromocresol green; alanine and aspartate aminotransferase: kinetic UV IFCC liquid). The body mass of each animal was monitored for 5 days after injection. To measure cell viability, 4T1 and ID8 cells were seeded at 2.4×10^3 cells per milliliter of expansion medium in 96-well plates by adding 100 µL of cell suspension to each well, followed by 24 h of incubation before replacing the medium with 100 µL fresh expansion medium, containing FRET ABN at concentrations 0.2 mg mL^{-1}, 0.4 mg mL^{-1}, 0.6 mg mL^{-1} and 0.8 mg mL^{-1}. The same volume of fresh medium with 25 mM acetaminophen was added to extra wells to serve as a positive control. The plates were incubated for 24 h before removing the medium and adding to all wells 250 µL of fresh medium with MTT tetrazolium dye at 1 mg mL^{-1}. Then they were incubated at 37 °C for 4 h before centrifuged at 700 *g* for 3 min. The medium was aspirated and replaced with 200 µL of DMSO followed by 25 µL of 0.1 M glycine buffer, 0.1 M NaCl and pH 10.5. The absorbance of each well was read at 570 nm (Varioskan TM LUX 3020-197, Thermo Fisher Scientific).

Expression Levels of MMP-2 in the 4T1 Mammary Tumor Model

To confirm MMP-2 expression in the mouse breast tumor model, standards methods for Western blotting were used for the detection of MMP-2 from mammary tumor lysates. Breast tumors harvested from the fourth mammary fat pad and healthy breasts were homogenized in five volumes of PBS relative to the mass using a Potter tissue homogenizer and spun down at 10,000 rpm for 20 min at 4 °C. The supernatants were further homogenized in 25 mM Tris-Cl buffer, containing 2 M urea and 0.5% trizol reagent by sonicating for 12 s at an amplitude of 35% before total protein quantification by the Bradford assay (Bio-Rad). Blots were produced using 12% polyacrylamide gel to resolve 15 µg of total protein. Gels were run at ~200 V for 60 min before transferring onto nitrocellulose membrane at 70 V for 90 min. The membranes were then blocked for 1 h before being incubated with mouse antihuman MMP-2 (Merck, MAB 13406, 1:1,000) and rabbit antimouse GAPDH (Thermo Fisher Scientific, PA1-987, 1:5,000) for 12 h at 4 °C. Antibody bindings were detected using rabbit antimouse IgG (Abcam, ab190475, 1:5,000) and chemiluminescence labeled goat antirabbit IgG

(Abcam, ab6721, 1:5,000) for 2 h at room temperature before imaging (ChemiDoc Touch, Bio-Rad). Before performing Western blot experiments, the identity and similarity of the human and mouse MMP-2 sequence were searched using Clustal Omega, suggesting a cross-reaction between antihuman MMP-2 antibody and mouse MMP-2.[1]

Pharmacokinetic Studies

Six to eight-week-old female C57BL/6 mice (Multidisciplinary Center for Investigation/Unicamp) were injected through the tail vein with the ABN (200 μL, 3 μM) in phosphate buffer (5 mM Na_2HPO_4 and pH 7.2) or with free peptides (200 μL, 2.4 μM) in PBS. The blood was drawn from the subclavian vein of 3 to 5 living mice in each set and at different time points for 24 h. The blood was transferred to a microcentrifuge tube filled with 10 μL of 3.2% citrate solution (m/v) until the final volume of 100 μL. The samples were spun down at 2,500 rpm for 15 min at 8 °C. Plasma was diluted 5-fold in PBS before fluorescence spectra were recorded after excitation at 496 nm and 669 nm to measure ABN, peptide, and synthetic biomarker signals. Four mice intravenously injected with free peptides (200 μL, 2.4 μM) in PBS were placed in individual cages for urine collection at different time points over 24 h. And samples were diluted 10-fold in PBS before fluorescence recording. Urinary enrichment was estimated by considering the mean plasma volume of 1.5 mL in 20–22 g female mice.

Breast Cancer Orthotopic Model Studies

Forty microliters of a suspension at 2.5×10^5 of 4T1 cells per mL in PBS were unilaterally injected into the fourth mammary fat pad of twelve-to-fourteen-week-old female BALB/c mice (Ribeirão Preto Central Animal Facility/USP). Control group mice were injected with 40 μL of PBS only. Tumor volume (V_T) was $V_T = \frac{1}{2}\left(L \times W^2\right)$, where length ($L$) was the larger dimension and width (W) the smaller one. All mice were injected through tail vein with the ABN (150 μL, 5 μM) in phosphate buffer 28 days after tumor cell or sham injections, and the experiments were carried out 0.25 h, 0.5 h, 1 h, 2 h, 4 h, 8 h and 16 h later for biodistribution measurements and urinalysis. At each time point, blood and urine

were sampled before transcardiac perfusion with PBS at 37 °C to remove blood from the circulation and then the major organs and tumor were harvested. The blood was drawn from the subclavian vein and placed in tubes filled with 20 µL of citrate solution to reach a final volume of 200 L before centrifugation to collect plasma that was diluted 5-fold in PBS, and fluorescence signals were recorded. Urine was collected by aspiration from the bladder through a needle. Then was diluted 10 to 20-fold in PBS, and fluorescent signals were recorded using a fluorimeter. The total protein content in urine samples was quantified by the Bradford assay (Bio-Rad) before separation by 17.5% polyacrylamide gel electrophoresis. Gels were run at 200 V for 60 min and excited at 488 nm and 647 nm before being imaged (Versadoc Imaging System 1000, Bio-Rad). After perfusion; spleen, liver, kidneys, lungs, heart, and tumor were harvested, flash frozen in liquid nitrogen and stored at –80 °C until further processing. Organs and tumors were homogenized in cold PBS at volumes five times greater than the equivalent volume of their masses using a Potter homogenizer before centrifugation at 10,000 rpm for 20 min at 8 °C. The supernatants were collected and the fluorescence spectra were recorded at excitation wavelength 669 nm and emission wavelength 686 nm to determine levels of accumulated ABN.

Preparation of a Paper-Based Lateral Flow Assay

The two test lines were printed (Image Technology, Isoflow Dispenser) on a nitrocellulose membrane (Sartorius, Unisart CN140) using a solution of streptavidin (Roche, 11721674001) at 1 mg mL^{-1} in phosphate buffer (5 mM Na_2HPO_4 and pH 7.2) along with a control line printed with goat antimouse IgG (EMD Millipore, AQ127) at 1 mg mL^{-1} in phosphate buffer (0.01% Na_3PO_4, 0.25 M NaCl and pH 7.6) and allowed to dry under controlled conditions in a chamber for 12 h. The membrane was then placed on an adhesive backing card before placing an absorbent pad (Merck Millipore, CFSP13000) on the distal end of the strip; and a sample pad (Cytiva, Standard 14) on the opposite one. The assembly was cut (Kinbio, ZQ 2002) in strips 25 mm long and 4 mm wide. Nanoparticle-antibody conjugates were prepared using forty-nanometer-gold nanoparticles (Abcam, ab154873) and human recombinant monoclonal fluorescein antibodies (Abcam, ab206509) according to the manufacturer’s instructions. To remove the

unbound antibodies, 450 µL of quencher diluted 10-fold in water was added to the conjugate mix before centrifugation at 9,000 *g* for 10 min at room temperature. The supernatant was removed and the conjugates suspended in diluted quencher with the addition of 0.1 to 0.4% (m/v) of bovine serum albumin (BSA). The conjugate solution was stored at 4 °C before use. All microfluidic assays were performed placing the strips into a separate microcentrifuge tube filled with synthetic biomarker solutions [phosphate buffer (5 mM, pH 7.2 and 1% (m/v) of BSA] to be immunochromatographed for 15 to 30 min, depending on the room conditions. The strip was moved to a resting surface to dry for 5 min before pipetting 6 µL of conjugate solution onto the sample pad. The strip was then immersed in another tube filled with 200 µL of washing buffer [1% (m/v) Tween20 in PBS] until the color developed in the test and control lines, and allowed to dry. For all experiments, strips were imaged with a Galaxy A14 5G phone camera and all images were analyzed with ImageJ, in which they were converted to 16-bit gray scale, rectangles surrounding the test and control lines were drawn using Gel Analyzer plugin to plot pixel intensity profiles, and a wand tracing tool was used to quantify test line intensities.

<u>Statistical Analysis</u>

Statistical analyses were performed using PSPP (GNU, 1.6.2). Mean comparisons were performed with the t-test and one-way analysis of variance (ANOVA). *P* values less than 0.05 were considered to be significant. Sample sizes, post hoc statistical tests, and reproducibility of experiments are detailed in the figure legends.

Nanocarrier Size and Surface Charge Distributions

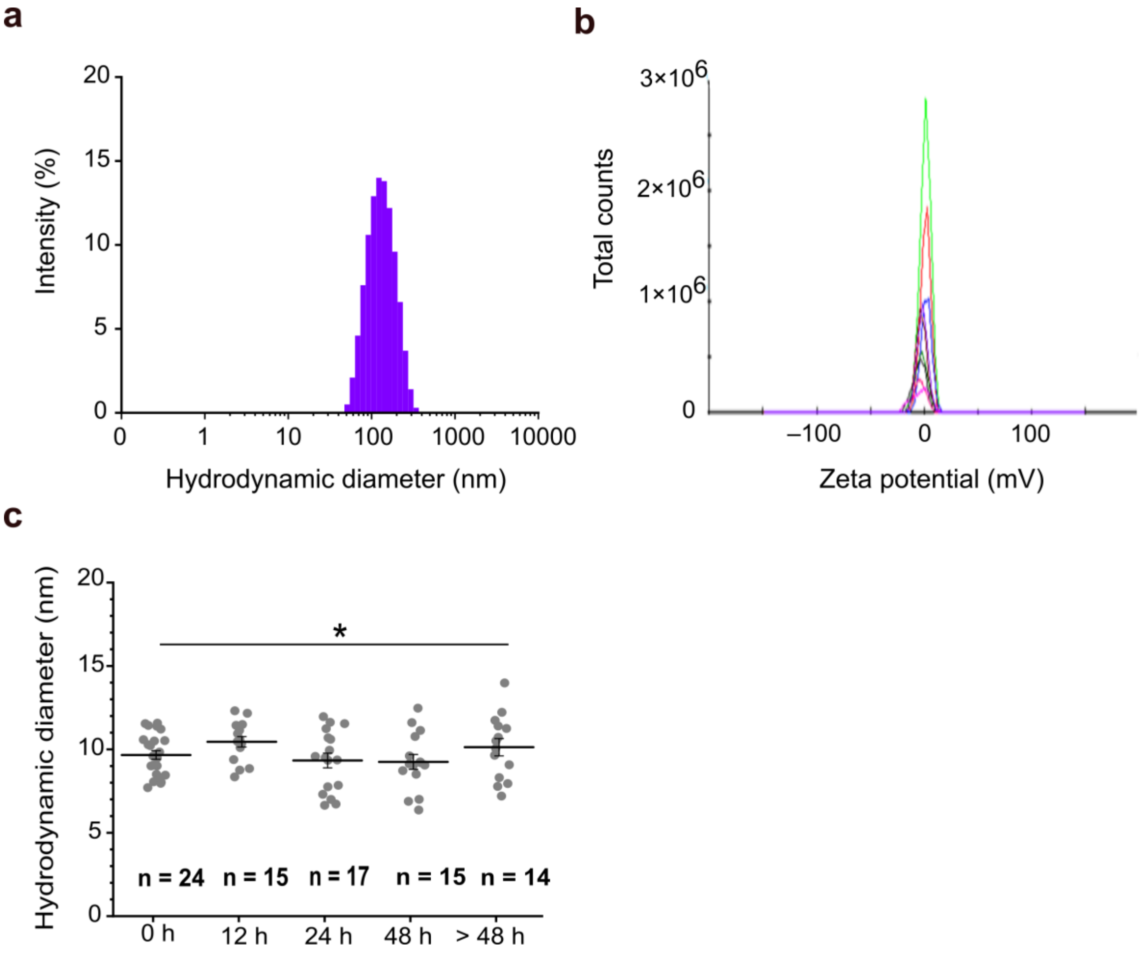

Figure S1. Nanocarrier size distribution and surface charge by electrophoretic mobility and stability by dynamic light scattering. (a) Histogram of size distribution weighted by intensity of dextran 150 kDa at 2 mg mL^{-1} in PBS with pH 7.4 at 37 °C used to validate procedure.[2] Experiments were completed independently three times, using aliquots from different batches. **(b)** Distribution of zeta potential measured by electrophoresis, indicating that the nanocarrier display a neutral surface charge with a propensity for agglomeration and precipitation.[3] Experiments were executed using dispersions at 2 mg mL^{-1} in saline (10 mM NaCl and pH 6.5) at 25 °C. **(c)** Volume-weighted hydrodynamic diameter measurements over time in dispersions at 2 mg mL^{-1} in PBS, showing good stability with no tendency to agglomerate 48 h after preparation. Three dispersions were prepared for each time point; Kruskal-Wallis H with Games-Howell post hoc test with correction for multiple comparison; $*P > 0.05$. Horizontal bars represent mean values.

Aberrant MMP-2 Activity in Tumors as Target

MMP-2 activity aids in angiogenesis by creating a defect in the extracellular matrix, easing the formation of blood vessels by endothelial cells in the surroundings of cancer cells.[4,5] Aberrant MMP-2 activity on the extracellular matrix can also result in degradation products, leading to signals of cell survival and migration; assisting tumor cells to intravasate into the vasculature; and activating invasion/metastasis.[6] Detection of intratumoral MMP-2 activity is challenging as ABNs have to identify a specific activity from among proteases of different catalytic classes after they extravasate the tumor vasculature to generate a detectable signal above any background. To identify MMP-2 as a sensible target, we considered a previous study that showcased MMP-2 and MMP-9 activities in a breast cancer mouse model by magnetic resonance imaging using nanoparticle-based sensors with chelated gadolinium conjugated onto the nanosensor surface.[7]

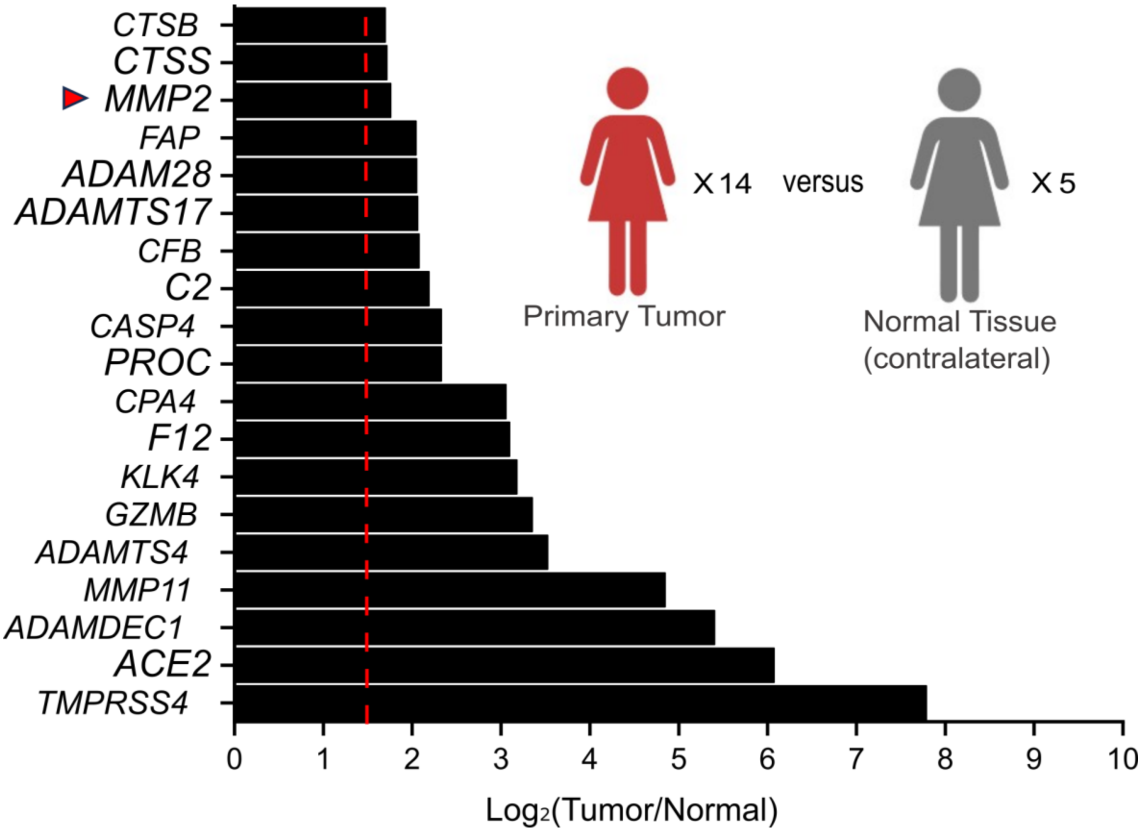

Figure S2. Differentially expressed protease genes in human triple negative breast cancer. Fold change analysis (Tumor/Normal) of protease genes derived

from a differential gene expression analysis of a bulk RNA sequence dataset available at the National Center for Biotechnology Information with GEO accession number GSE167152.[8] The red arrowhead indicates *MMP2* among other potential proteases to identify as a target. The dotted line is at Log2 (Tumor/Normal) equal to 1.5.

Synthesis of a Protease-Sensitive Nanosensor

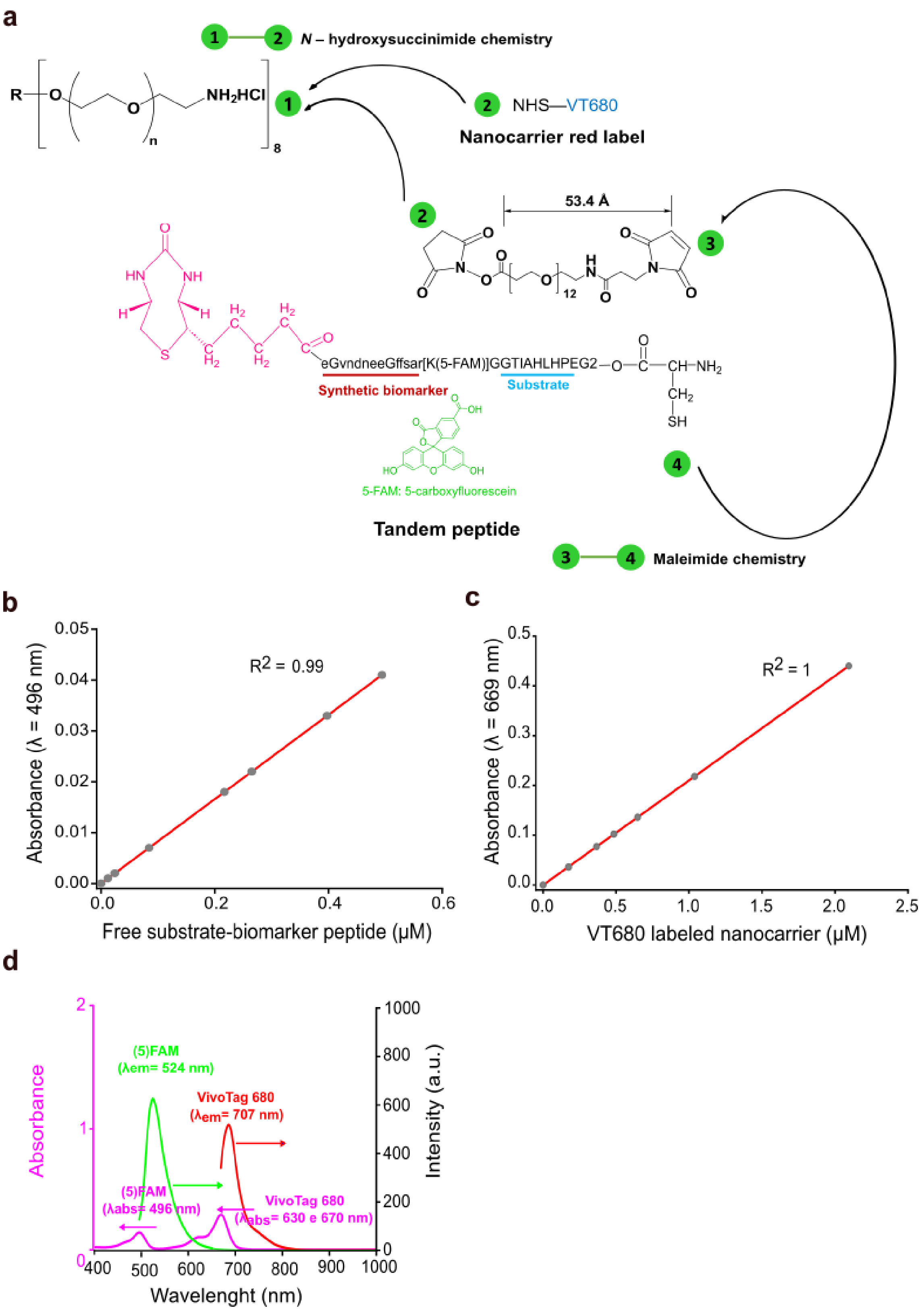

Figure S3. Synthesis of a protease-sensitive nanosensor via chemistry of *N*-hydroxysuccinimide and maleimide reactions, and the measurements of

valencies by spectrophotometry. **(a)** Schematic depicting the two-step construction of MMP-2 ABN based on the bioconjugation chemistry through *N*-hydroxysuccinimide (NHS) to conjugate dyes and maleimide-bearing crosslinkers onto the ABN surface. Maleimide group is targeted by the sulfhydryl group of cysteine in the tandem peptide to complete the ABN construction. The substrate was identified through in vitro selection in a controlled medium and not in a complex environment that could resemble the protease activity of blood. As such, it was typified by exhibiting the highest observed catalytic efficiency (1.1×10^4 $M^{-1} s^{-1}$) towards MMP-2. The substrate is also specific for MMP-15 to -17 and MMP-24 and -25 with lower catalytic efficiencies. MMP-9 and MMP-14 have no activity on it.[9] R, hexaglycerol core**.** The calibration curve for the photometry analysis of **(b)** peptide and **(c)** VT680 conjugation. Standards were prepared by spiking known quantities of red dyes and free peptides in PBS to quantify the amounts of molecules on the ABN surface. The amount of nanocarrier molecules was determined by synthesis mass balance calculations. **(d)** Fluorescence spectra of ABN confirming the activity of both dyes by showing maxima in the green and near infrared regions.

Paper-Based Lateral Flow Assay for Non-invasive Biomarker Detection

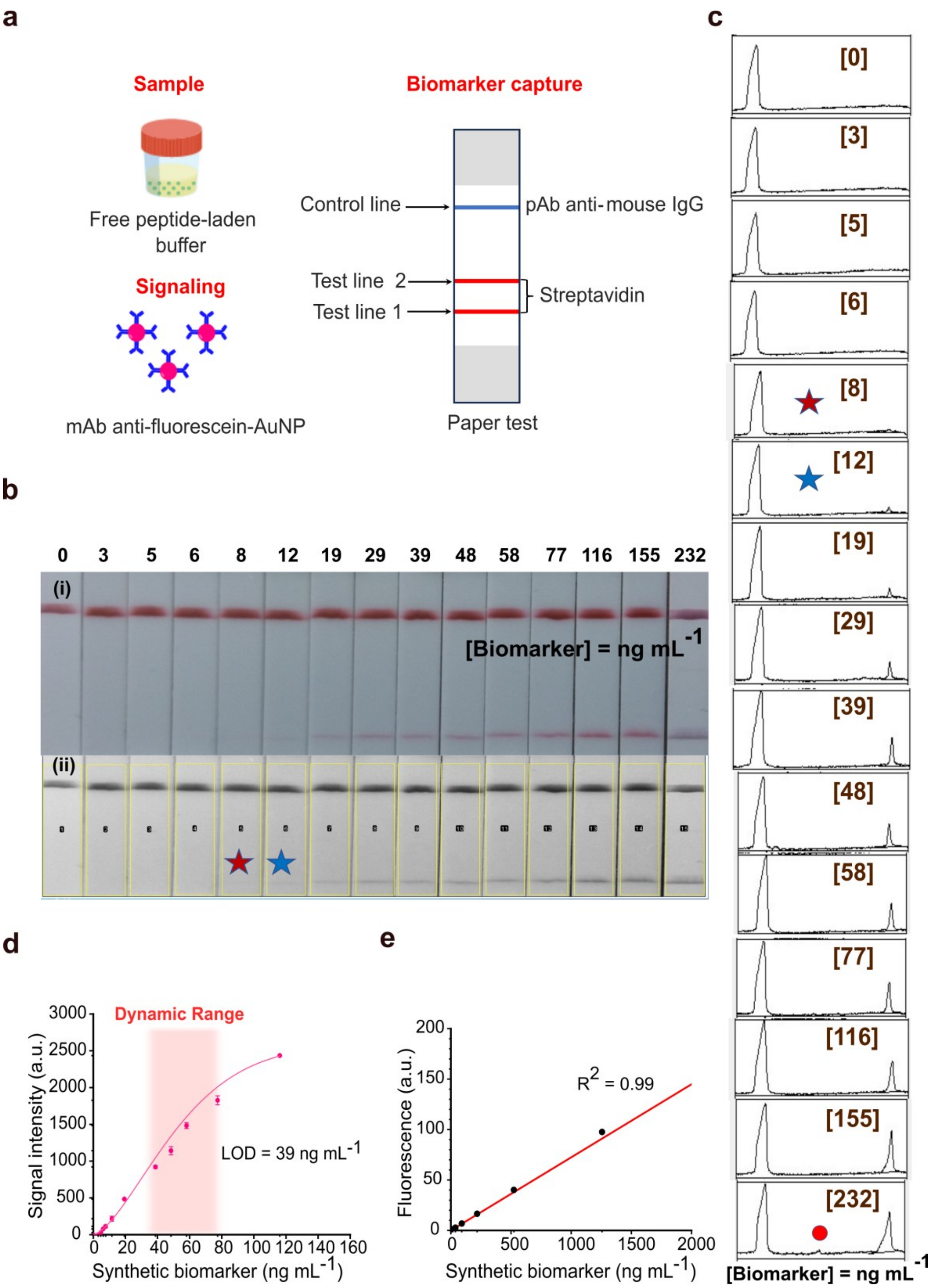

Figure S4. Rapid test for synthetic biomarker detection. (a) Schematic showing the items necessary to run a paper test to detect the activity biomarker

in urine. mAb, monoclonal antibody; AuNP, gold nanoparticle; pAb, polyclonal antibody; IgG, immunoglobulin G. (**b)** Image of test strips (i) acquired after a run to construct the calibration curve. The image of test strips (ii) converted to a 16-bit grayscale image, and each strip showing rectangular region (yellow) surrounding the test and control lines to determine the pixel intensity by generating peak areas **(c)** using the wand tracing tool. The red star indicates the minimum concentration at which the test line was visible by the naked eye, and the blue star indicates the minimum concentration for quantification. The red circle in the peak-area plot indicates at which concentration the first test line was saturated and the second-line color development started. **(d)** The Weibull function type 2 was fitted to the experimental data (mean ± s.e.m. is denoted by a horizontal line with error bars. $n = 2–3$; $R^2 = 0.99$). The dynamic range is highlighted, and the limit of detection (LOD) was determined considering the minimum concentration at which the coefficient of variation was less than 20%. **(e)** The correlation curve of the test line intensity and the biomarker concentration was confirmed by spectrophotometry analysis.

Probe Synthesis, Optical Characterization and MMP-2 Activity Assay

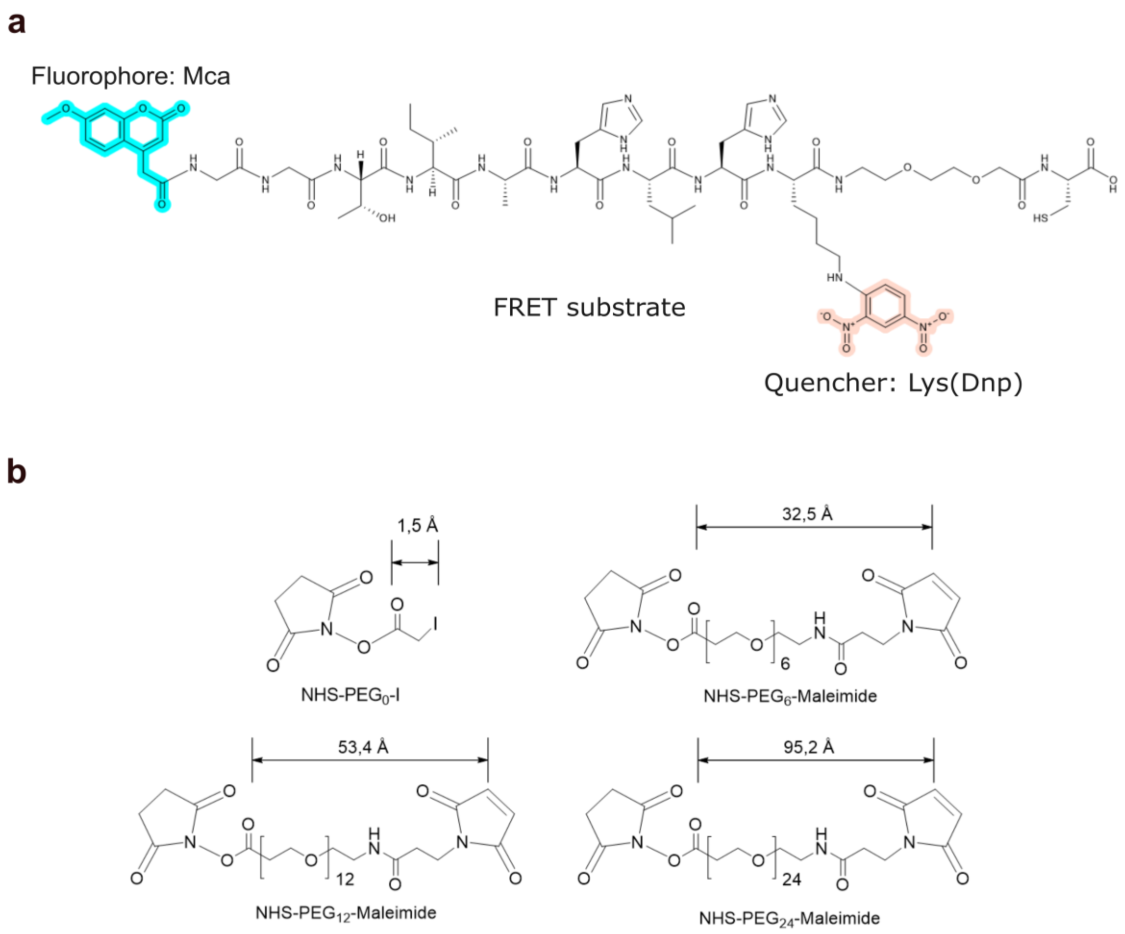

Figure S5. Synthesis of fluorogenic probes with varied cross-linker lengths. **(a)** Substrate molecular structure, highlighting the FRET pair. Mca, 7-methoxycoumarin-4-acetic acid; FRET, fluorescence resonance energy transfer; Lys(Dnp), 2,4 dinitrophenyl-lysine. **(b)** Different lengths of cross-linkers were used to synthesize fluorogenic probes for biomarker kinetic release assays. NHS, N-hydroxysuccinimide; PEG, poly(ethylene glycol); I, succinimidyl iodoacetate.

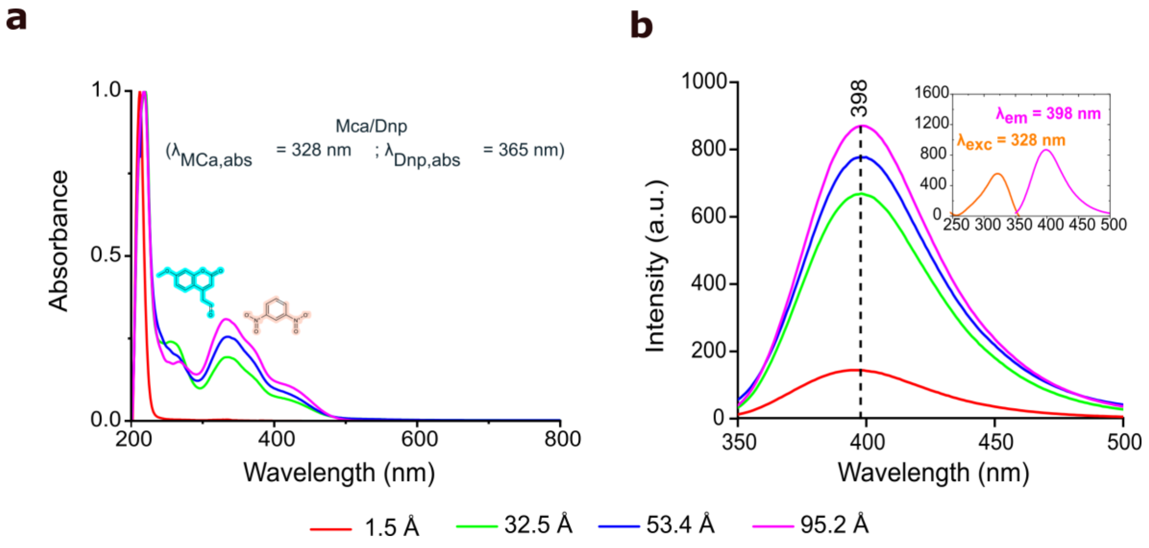

Figure S6. Fluorogenic FRET probe optical characterization. (a) UV-visible spectra recording using dispersions of the four probes, synthesized with a substrate flanked by the Mca/Dnp FRET pair and conjugated onto the nanocarrier surface with cross-linkers of varying lengths, in the assay medium immediately post synthesis. Mca, 7-methylcoumarin-4-acetic acid; Dnp, 2,4 dinitrophenyl group; abs, absorption. **(b)** Fluorescence emission spectra, highlighting the wavelength of maximum emission across all four probes and showing that longer cross-linkers yield probes with a higher degree of bioconjugation. exc, excitation; em, emission.

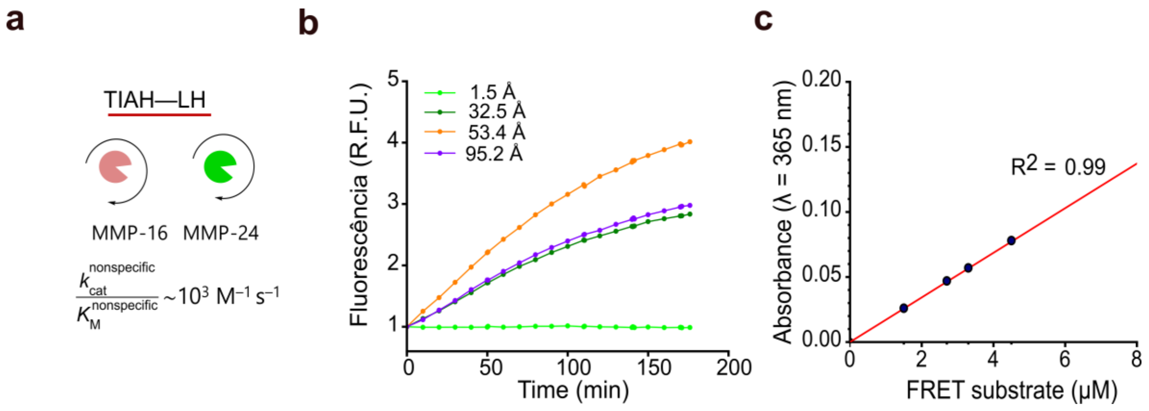

Figure S7. Enzymatic assay for Michaelis-Menten analysis of the catalytic efficiency of MMP-2. (a) Observed catalytic efficiency of MMP-16 and MMP-24, both considered potential nonspecific activities on the substrate TIAH–LH immobilized on the surface of microplate wells and incubated with 50 nM active proteases for 2 h at 37°C. The catalytic efficiency did not differ from the values estimated when the substrate was conjugated onto the nanocarrier surface at 53.4 Å.[9] K_{cat}, catalytic constant; K_M, Michaelis-Menten constant. **(b)** Kinetic fluorescence curves of fluorogenic probes at 3 μM during incubation with 32 nM MMP-2 for 3 h at 37 °C. The FRET probe with substrate at a distance of 1.5 Å from the core developed no detectable relative fluorescence, while fluorescence varied across probe concentrations when the substrate was conjugated at 95.2 Å from the nanocarrier core, causing linearity loss of initial cleavage rates. R.F.U., relative fluorescence unit. **(c)** Calibration curve built using aliquots of FRET peptide stock solution at 5 mg mL^{-1} to confirm the concentration of FRET substrate solutions by spectrophotometric analysis at 365 nm, using the lysine-2,4 -dinitrophenyl group [Lys(Dnp)] molar extinction coefficient.

FRET ABN Synthesis and Cytotoxicity Assessment

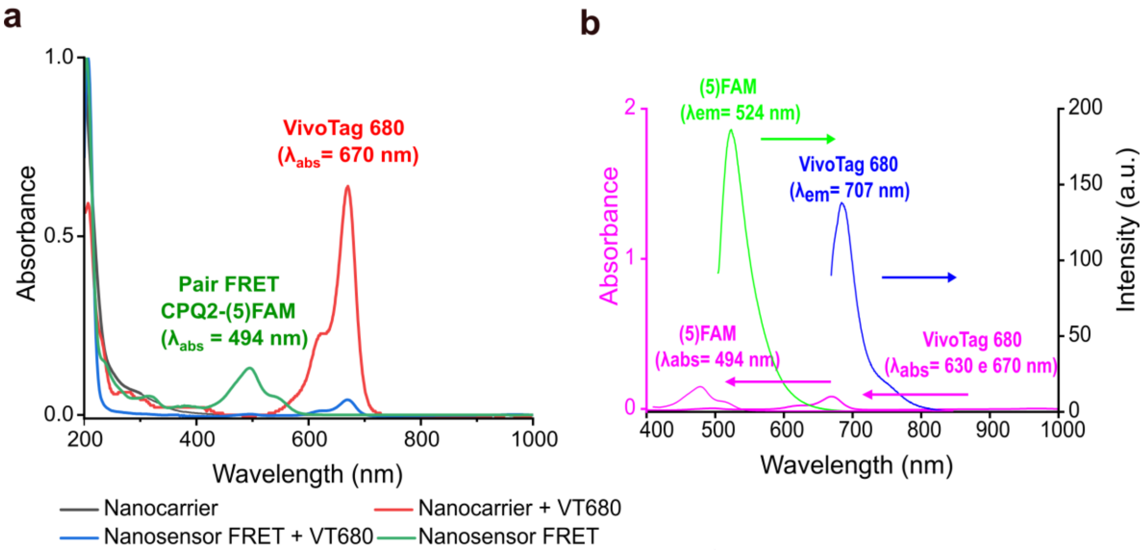

Figure S8. Optical characterization of FRET ABN functionalization for tumor cell cytotoxicity assays. (a) Spectra of nanocarrier dispersed in 10 mM phosphate buffer, containing 150 mM NaCl and pH 7.5 before peptide conjugation showing no absorption band. And of FRET ABN collected from a dispersion in 5 mM phosphate buffer with pH 7.2. **(b)** When VT680 is already on the nanocarrier surface, peptide conjugation is impaired and CPQ2 curtailed VT680 fluorescence emission through a mechanism different from FRET. For convenience, FRET ABN dispersions were used in the cytoxicity experiments to investigate the propensity of ABN to harm target organ cells. CPQ2, proprietary quencher; FRET, fluorescence resonance energy transfer.

ABN Biodistribution and Pharmacokinetic Studies

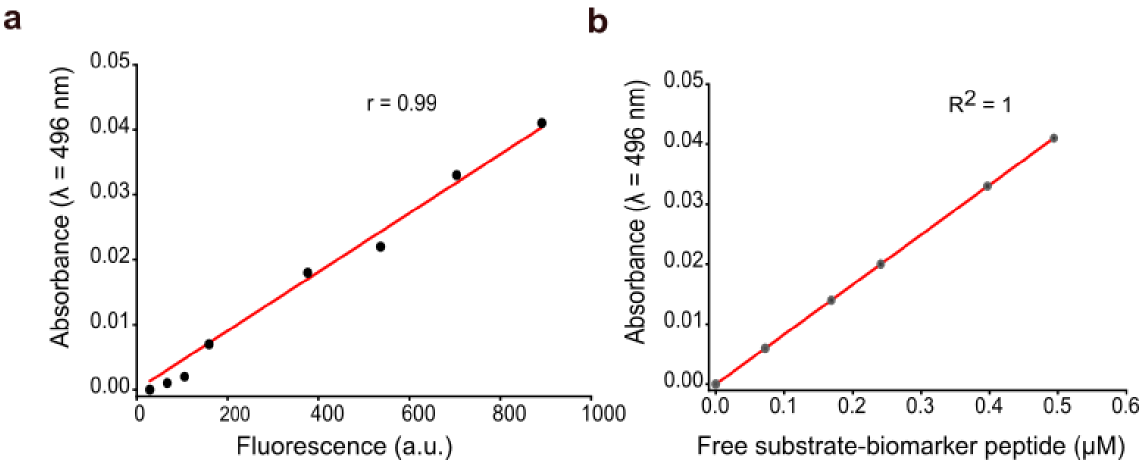

Figure S9. Quantification of synthetic biomarker in urine. (a) Signal correlation curve used for quantification of cleaved peptides. r, Pearson coefficient. **(b)** Built calibration curve for the quantification of the activity-based biomarker. Standards were prepared using urine from uninjected mice diluted 10-fold in PBS and spiked with known amounts of free peptides.

Mammary Tumor Model Studies

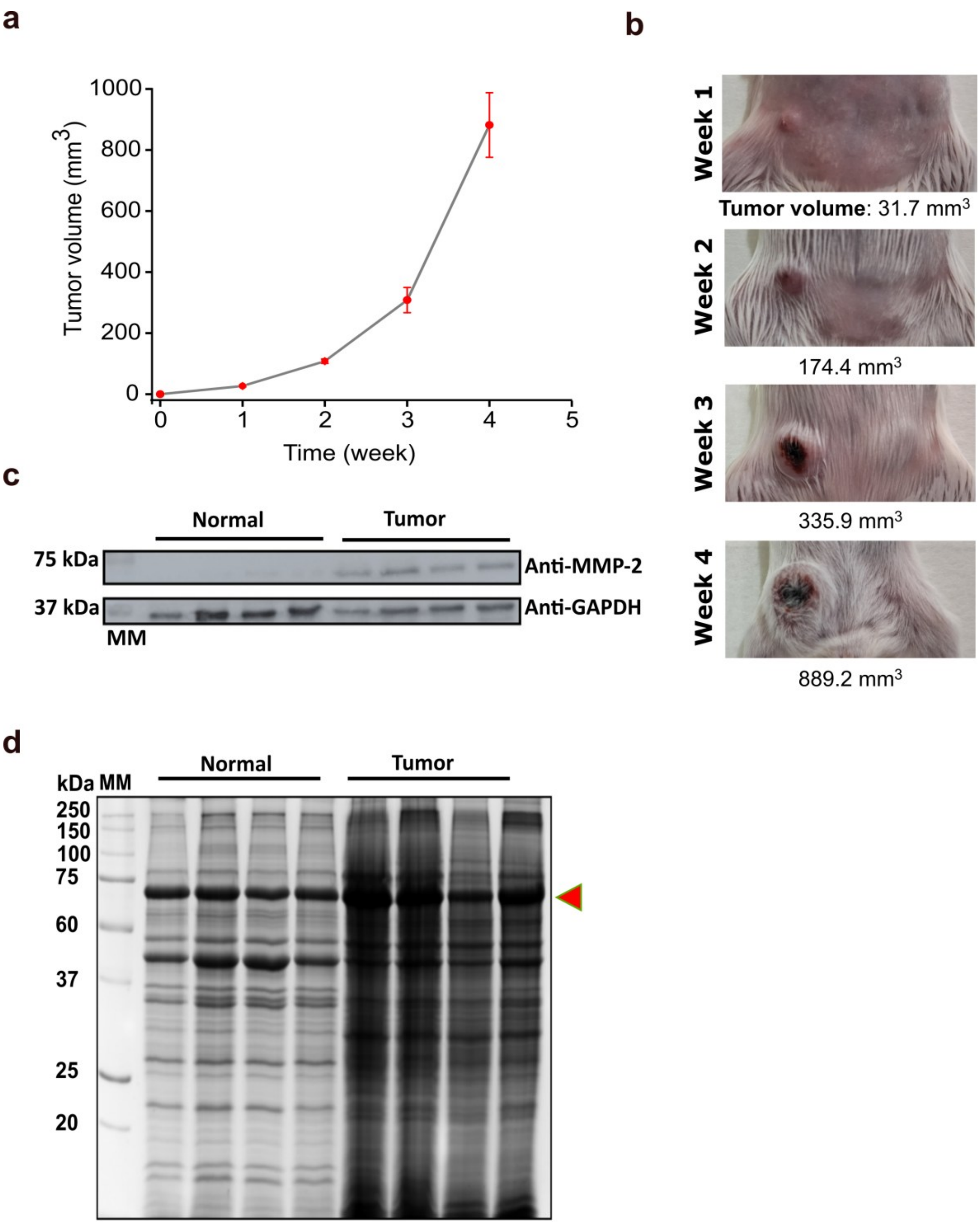

Figure S10. Growth of the orthotopic breast cancer model and its MMP-2 expression levels. (a) Profile of the total burden after 4T1 cell injection estimated by measuring tumor size using a digital caliper. mean ± s.e.m. is denoted by a horizontal line with error bars; n = 6 independent mice for all points. **(b)** Representative images of the breast tumor at each measurement time point.

(c) Western blot analysis of anti-MMP-2 in lysates displays a specific band detected for MMP-2 at a molecular weight of 75 kDa. Knowing that no band for MMP-2 was indicated at a molecular weight of 50 kDa (data not shown), it was assumed that MMP-2 in the tumor was activated by oxidation without losing the pro-domain, acquiring a new conformation that no longer blocks the catalytic domain.[10] The anti-MMP-2 antibody can recognize the zymogen and enzyme active forms. $n = 4$ mice per condition. GAPDH, glyceraldehyde-3-phosphate dehydrogenase; MM, molecular weight size marker. **(d)** Immunoblot of the gel in **(c)** for mouse MMP-2. Red arrowhead shows the approximate location of the MMP-2 band.

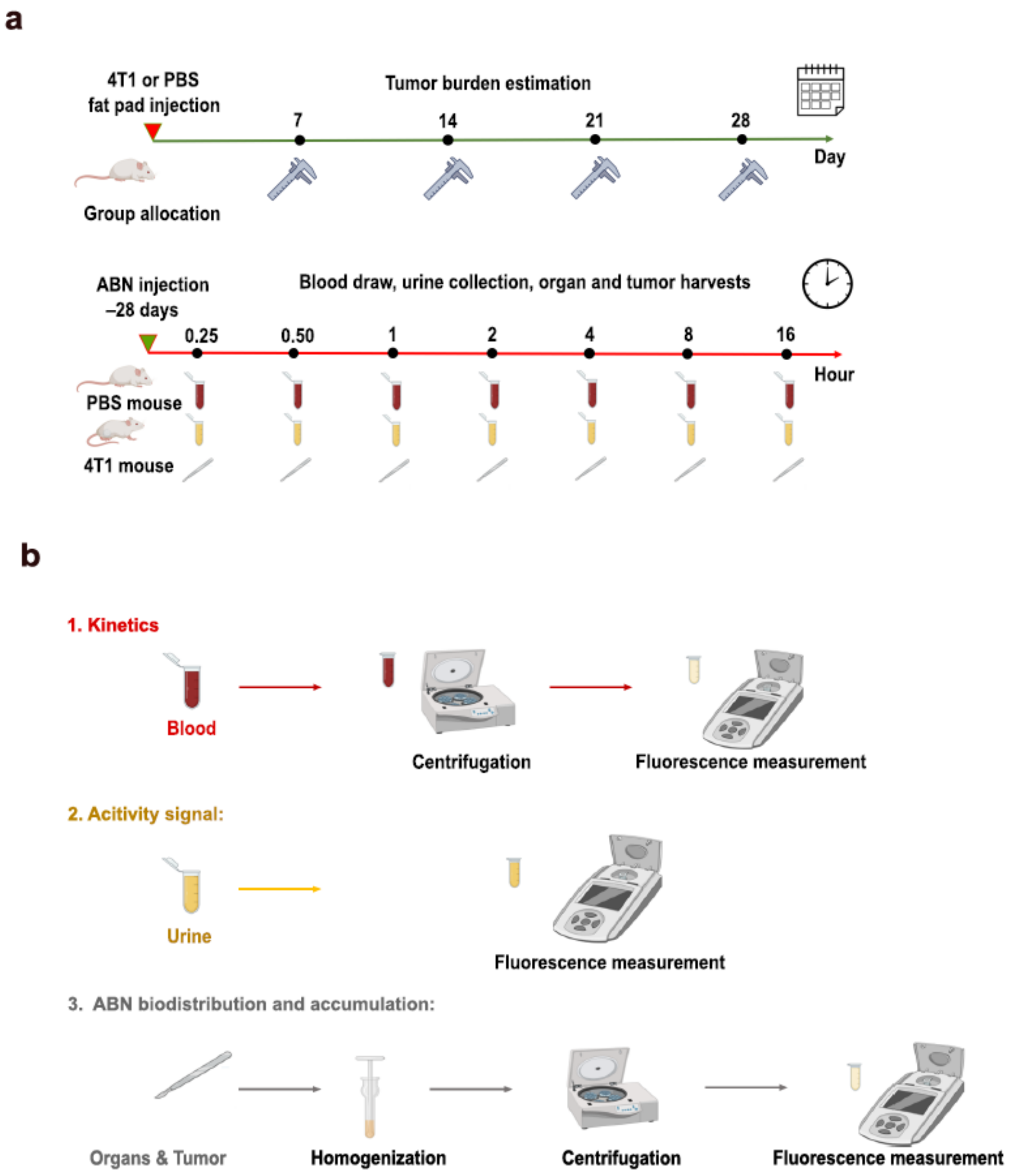

Figure S11. Work flow of sample preparation and measurements to assess ABN plasma kinetics, biodistribution, tumor accumulation, and activity signal. (**a**) Experimental approach to quantifying the pharmacokinetics and biodistribution of ABN and synthetic biomarker. Timeline of breast tumor measurements of total burden and post-injection urine collections, blood draws, and major organs and tumor harvests. (**b**) Plasma was diluted 5-fold in PBS before measurements. The urine samples were diluted 10- to 20-fold in PBS. The supernatant fluorescence of organ and tumor homogenates was measured with no sample dilution. All measurements were performed in a cuvette.

a

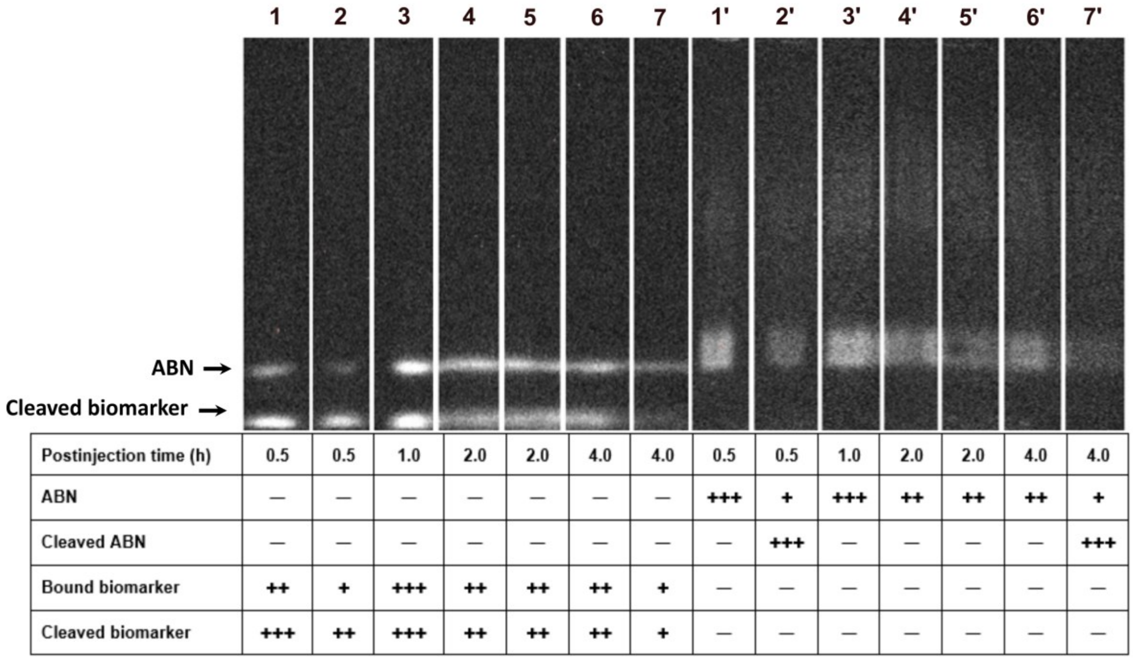

Postinjection time (h)	0.5	0.5	1.0	2.0	2.0	4.0	4.0	0.5	0.5	1.0	2.0	2.0	4.0	4.0
ABN	—	—	—	—	—	—	—	+++	+	+++	++	++	++	+
Cleaved ABN	—	—	—	—	—	—	—	—	+++	—	—	—	—	+++
Bound biomarker	++	+	+++	++	++	++	+	—	—	—	—	—	—	—
Cleaved biomarker	+++	++	+++	++	++	++	+	—	—	—	—	—	—	—

b

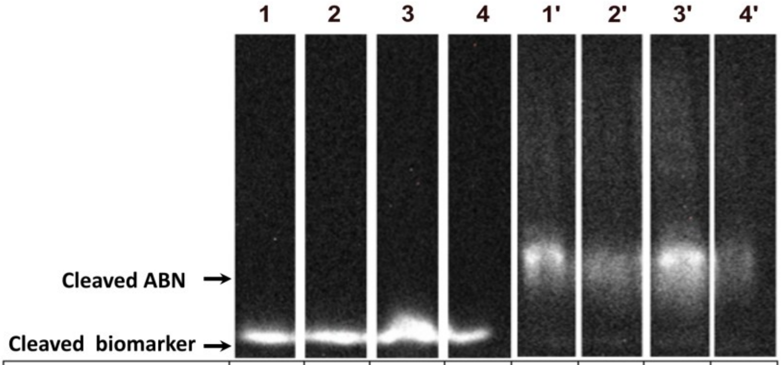

Postinjection time (h)	0.5	1.0	1.0	2.0	0.5	1.0	1.0	2.0
ABN	—	—	—	—	—	—	—	—
Cleaved ABN	++	+	++	+	—	—	—	—
Bound biomarker	—	—	—	—	—	—	—	—
Cleaved biomarker	—	—	—	—	+++	+++	+++	+++

Figure S12. Fluorescent visualization of ABN cleavage by tumor-associated proteases through urine separation by polyacrylamide gel electrophoresis. (a) Fluorescent visualization of gels after separation of urine collected from healthy mice, indicating the presence of ABN at postinjection time points. **(b)** Fluorescent visualization of gels after separation of urine from tumor-bearing mice, indicating the presence of cleaved ABN at different time points. The gels were run according to the availability of urine samples. While lane groups 1–7 and 1–4 were excited at 488 nm for visualization of the bound synthetic biomarker band (higher band) and cleaved (lower band), lane groups 1’–7’ and 1’–4’ were

excited at 647 nm for visualization of the label nanocarrier band. The attribution of the **+** and **–** symbols was for qualitative purposes to help identify representations.

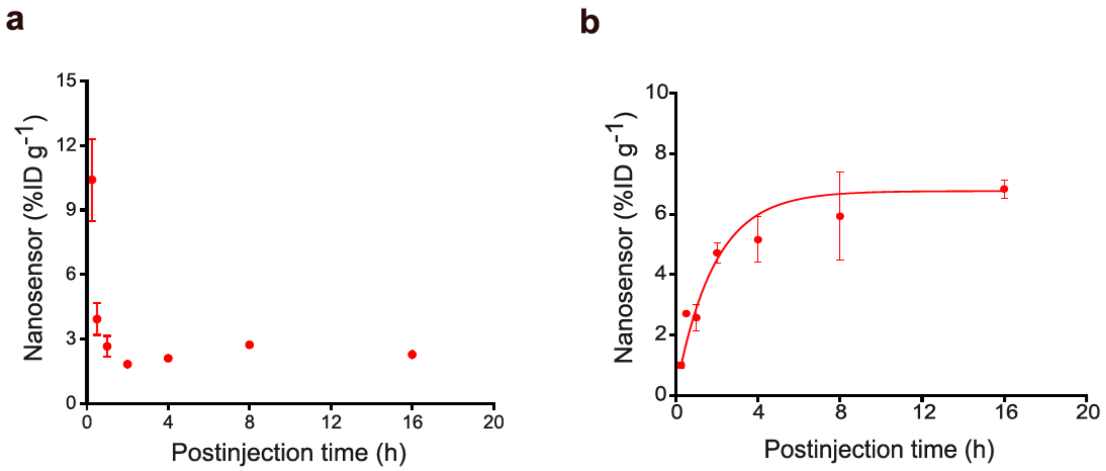

Figure S13. Nanosensor accumulation in tumor-bearing mice organs. (a) ABN accumulation in kidneys, showing a decay over time. mean ± s.e.m. is denoted by a horizontal line with error bars; $n = 2–4$ mice. **(b)** ABN accumulation in spleen, revealing a saturation profile. mean ± s.e.m. is denoted by a horizontal line with error bars. Line denotes the fit of one-phase exponential growth function with time constant parameter to the data ; $n = 2–4$ mice; $R^2 = 0.99$.

Discussion of DNA Blood Testing for Cancer Screening

Table S1 – Results of prospective studies on blood DNA testing for multicancer detection, highlighting the test performance at early stages.

**Test name**	GRAIL	DELFI	CancerSEEK
**Number of cancers**	12 (50){16}	7	8 (17)
**Biomarker**	Methylation signature	DNA fragmentation and mutations	DNA mutations and specific-cancer proteins
**Test result**	Cancer and DNA origin	Cancer and location	Cancer and location
**Performance**	Specificity: 99.3% {98.4%}<br>Sensibility: 67.3% (43.9%) {66.3%}<br>TOO: 93% {85.2%}	Specificity: 98%<br>Sensibility: 73%<br>TOO: 75%	Specificity: >99% (98.9%)<br>Sensibility: 70% (27%)<br>TOO: median 63%
**Sensitivity for early detection**	Stage<br>I: 39%(18%){24.2%}<br>II: 69%(43%) {–}	Stage<br>I: 68%<br>II: 72%	Stage<br>I: 43% (–)<br>II: 73% (–)
**References**	Liu *et al.*, ***Ann. Onc.***, 2020 [11]<br>Nicholson *et al.*, ***Lancet,*** 2023 [12]	Cristiano *et al.*, ***Nature***, 2019 [13]	Cohen *et al.*, ***Science***, 2018 [14]<br>Lennon *et al.*, ***Science***, 2020 [15]

TOO, Tissue of origin; DELFI, DNA evaluation of fragments for early detection.

Supporting References

1. Sievers, F. *et al.* Fast, scalable generation of high-quality protein multiple sequence alignments using clustal omega. *Mol. Syst. Biol.* **7**, 1–6 (2011).
2. ASTM. Standard Test Method for Measuring the Size of Nanoparticles in Aqueous Media Using Dynamic Light Scattering. *ASTM Int.* 1–22 (2021).
3. ASTM. Standard Guide for Measurement of Electrophoretic Mobility and Zeta Potential of Nanosized Biological Materials. *ASTM Int.* 1–7 (2022).
4. Kessenbrock, K., Plaks, V. & Werb, Z. Matrix metalloproteinases: regulators of the tumor microenvironment. *Cell* **141**, 52–67 (2010).
5. Stetler, W. G. Matrix metalloproteinases in angiogenesis a moving target for therapeutic intervention. *J. Clin. Invest.* **103**, 1237–1241 (1999).
6. Hanahan, D. & Weinberg, R. A. Hallmarks of cancer: the next generation. *Cell* **144**, 646–674 (2011).
7. Olson, E. S. *et al.* Activatable cell penetrating peptides linked to nanoparticles as dual probes for in vivo fluorescence and MR imaging of proteases. *Proc. Natl. Acad. Sci. U. S. A.* **107**, 4311–4316 (2010).
8. Kim, T. *et al.* Comparative characterization of 3D chromatin organization in triple-negative breast cancers. *Exp. Mol. Med.* **54**, 585–600 (2022).
9. Ratnikov, B. I. *et al.* Basis for substrate recognition and distinction by matrix metalloproteinases. *Proc. Natl. Acad. Sci. U. S. A.* **111**, E4148–E4155 (2014).
10. Sariahmetoglu, M. *et al.* Regulation of matrix metalloproteinase-2 (MMP-2) activity by phosphorylation. *FASEB J.* **21**, 2486–2495 (2007).
11. Liu, M. C. *et al.* Sensitive and specific multi-cancer detection and localization using methylation signatures in cell-free DNA. *Ann. Oncol.* **31**, 745–759 (2020).

12. Nicholson, B. D. *et al.* Multi-cancer early detection test in symptomatic patients referred for cancer investigation in England and Wales (SYMPLIFY): a large-scale, observational cohort study. *Lancet Oncol.* **24**, 733–743 (2023).

13. Cristiano, S. *et al.* Genome-wide cell-free DNA fragmentation in patients with cancer. *Nature* **570**, 385–389 (2019).

14. Cohen, J. D. *et al.* Detection and localization of surgically resectable cancers with a multi-analyte blood test. *Sci. Mag.* **359**, 926–930 (2018).

15. Lennon, A. M. *et al.* Feasibility of blood testing combined with PET-CT to screen for cancer and guide intervention. *Sci. Mag.* **369**, 1–16 (2020).
